# Immunometabolic modulation of retinal inflammation by CD36 ligand

**DOI:** 10.1038/s41598-019-49472-8

**Published:** 2019-09-09

**Authors:** Katia Mellal, Samy Omri, Mukandila Mulumba, Houda Tahiri, Carl Fortin, Marie-France Dorion, Hung Pham, Yesica Garcia Ramos, Jinqiang Zhang, Sheetal Pundir, Jean-Sébastien Joyal, Jean-François Bouchard, Florian Sennlaub, Maria Febbraio, Pierre Hardy, Simon-Pierre Gravel, Sylvie Marleau, William D. Lubell, Sylvain Chemtob, Huy Ong

**Affiliations:** 1 Faculty of Pharmacy, Université de Montréal, Montreal, Canada; 2 Maisonneuve-Rosemont Hospital, Montréal, Canada; 3 Mperia Therapeutics, Montréal, Canada; 4 Department of Chemistry, Université de Montréal, Montreal, Canada; 5 Departments of Pediatrics, Ophthalmology and Pharmacology, Université de Montréal, Montreal, Canada; 6 Neuropharmacology Laboratory, School of Optometry, Université de Montréal, Montreal, Canada; 7 Institut de la Vision, Sorbonne Universités, INSERM, CNRS, Paris, France; 8 Department of Dentistry, University of Alberta, Edmonton, Canada

**Keywords:** Receptor pharmacology, Chronic inflammation

## Abstract

In subretinal inflammation, activated mononuclear phagocytes (MP) play a key role in the progression of retinopathies. Little is known about the mechanism involved in the loss of photoreceptors leading to vision impairment. Studying retinal damage induced by photo-oxidative stress, we observed that cluster of differentiation 36 (CD36)-deficient mice featured less subretinal MP accumulation and attenuated photoreceptor degeneration. Moreover, treatment with a CD36-selective azapeptide ligand (MPE-001) reduced subretinal activated MP accumulation in wild type mice and preserved photoreceptor layers and function as assessed by electroretinography in a CD36-dependent manner. The azapeptide modulated the transcriptome of subretinal activated MP by reducing pro-inflammatory markers. In isolated MP, MPE-001 induced dissociation of the CD36-Toll-like receptor 2 (TLR2) oligomeric complex, decreasing nuclear factor-kappa B (NF-κB) and NLR family pyrin domain containing 3 (NLRP3) inflammasome activation. In addition, MPE-001 caused an aerobic metabolic shift in activated MP, involving peroxisome proliferator-activated receptor-γ (PPAR-γ) activation, which in turn mitigated inflammation. Accordingly, PPAR-γ inhibition blocked the cytoprotective effect of MPE-001 on photoreceptor apoptosis elicited by activated MP. By altering activated MP metabolism, MPE-001 decreased immune responses to alleviate subsequent inflammation-dependent neuronal injury characteristic of various vision-threatening retinal disorders.

## Introduction

Inflammation is a critical component in degenerative retinal diseases independent of underlying pathological mechanism^1^. Influx, activation and accumulation of mononuclear phagocytes (MP; that comprise monocytes, microglia and macrophages^2^) into the subretinal space cause collateral tissue damage^1,3^. The ensuing inflammatory cascade is integral for the ultimate death of photoreceptor cells in progressive vision-threatening maladies, including retinitis pigmentosa, diabetic retinopathy and age-related macular degeneration^4,5^. In this context, injured tissues produce damage-associated molecular patterns (DAMPS) that activate Toll-like receptors (TLRs)^6,7^ upregulating and sustaining release of pro-inflammatory cytokines and chemokines, which propagate into chronic inflammation resulting in degeneration of retinal pigment epithelium (RPE) and photoreceptors^8^.

On the membrane surface of MP, the cluster of differentiation 36 receptor (CD36) is co-expressed with the TLR2/6 heterodimer assembly^9^. A scavenger of various debris, including oxidized lipids^8,10,11^, CD36 has been shown to sustain TLR2/6 signaling elicited by diacylglycerols^12^, and to regulate TLR2-dependent macrophage driven inflammation^13^. Following the canonical pathway of activation of TLR2/6-CD36 complex, MyD88 recruits the kinase IRAK4 that binds and phosphorylates IRAK1.The ensued formation of IRAK1 - TNF receptor associated factor 6 (TRAF6) complex incorporates transforming growth factor beta activated kinase (TAK1)^14^. TAK1 in turn activates NF-κB by IKK complex phosphorylation, as well as mitogen-activated protein kinases (MAPKs) P38, and c-Jun N-terminal kinase (c-JNK), promoting activation of various transcription factors, including AP-1, and cytokine production^14,15^. Notably, azapeptide analogs of growth hormone releasing peptide-6 (GHRP-6, H-His-D-Trp-Ala-Trp-D-Phe-Lys-OH) designed by us, exhibited *in vitro* low micro-molar CD36 binding affinity, possessed high selectivity, and inhibited nitric oxide produced by MP stimulated with the TLR2-agonist fibroblast-stimulating lipopeptide (R-FSL-1)^16^. Towards the development of therapy to mitigate degenerative retinal diseases, the role of CD36 has now been elucidated using pharmacologic and genetic approaches. In a mouse model of subretinal inflammation, the CD36 azapeptide modulator [azaY^4^]-GHRP-6 (MPE-001) has been evaluated and found to be a novel therapeutic prototype having a unique mode of action that curtails photoreceptor damage induced by relevant photo-oxidative stress. MPE-001 reduced markedly MP infiltration and the inflammatory cytokine profile in the subretinal space and preserved photoreceptor structural integrity and function. The effects of MPE-001 were CD36-dependent. In an unprecedented manner, MPE-001 modulated the inflammatory profile of MP by attenuating the inflammasome cascade. Since MP phenotype is regulated by cellular metabolism^17^, we tested and found that MPE-001 elicited a shift in metabolic pathways of M1-type MP from a glycolytic state to one favoring oxygen consumption, which in turn altered NLR family pyrin domain containing 3 (NLRP3) expression. Thus, immune-metabolic modulation by CD36 ligands, such as MPE-001, offers a promising new means for curbing chronic inflammation characteristic of degenerative eye diseases.

## Results

### MPE-001 represses subretinal inflammation and protects against photoreceptor degeneration *in vivo*

**Figure 1:**
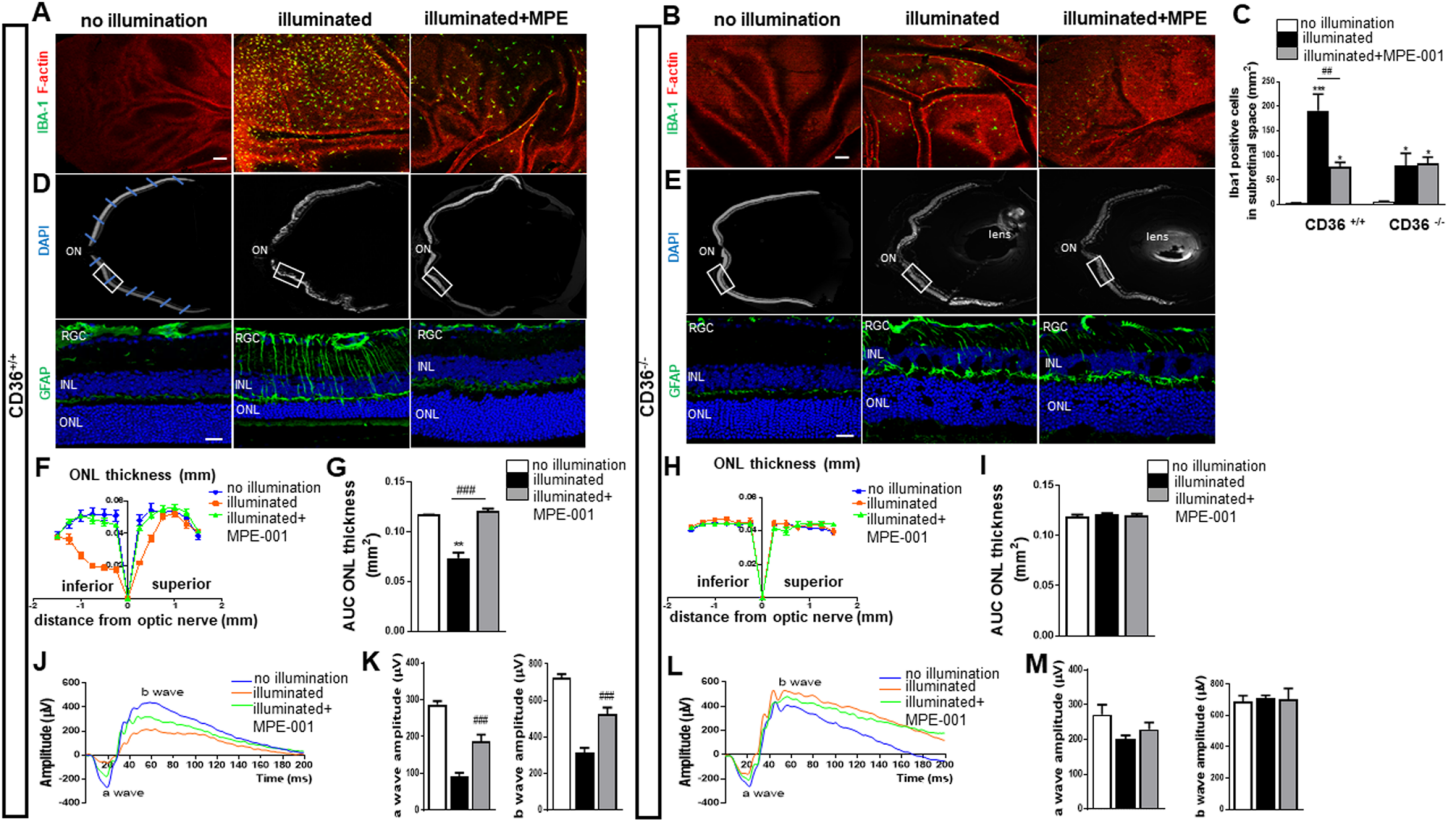
Azapeptide MPE-001 modulates subretinal MP accumulation and protects against photoreceptors degeneration and function. (**A**–**M**) CD36^+/+^ (n = 6 mice/group) and CD36^−/−^ mice (n = 3 mice/group) were illuminated for 5 days with 6000 lux blue light and subcutaneously injected with 289 nmol/kg per day of MPE-001, starting at 24 h following blue-light exposure for a total of 7 consecutive days. **(A**,**B)** F‐actin of RPE cells was counterstained with rhodamine phalloidin (red), showing IBA-1^+^ MPs (green) in the subretinal space of central retina from CD36^+/+^ and CD36^−/−^ mice. Scale bar: 25 μm. **(c)** IBA-1^+^ cell quantification in the subretinal space. **(D,E)** Upper panels show representative cryosections with nuclear staining (DAPI) of central retina with the optic nerve (ON) from CD36^+/+^ or CD36^−/−^ mice. The blue lines indicate the location of measurements of the ONL thickness on each side of the optic nerve (ON). Lower panels show representative images of central retina (12X magnification of white square) stained with GFAP (green). **(F,H)** Spider graphs of the ONL thickness measured at defined distances of the ON. **(G,I)** Bar graph showing ONL AUC from CD36^+/+^**(F,G)** and CD36^−/−^ mice **(H,I)**. **(J**–**M)** Representative scotopic ERG responses from CD36^+/+^ **(J)** and CD36^−/−^ mice **(l)**. Quantification of a and b wave amplitudes ERG from CD36^+/+^ **(K)** and CD36^−/−^ mice **(M)** at a light intensity of 3.0 cd*s/m². In **C**,**G**,**K**,**M** one-way ANOVA test with Newman-Keuls post-test for multiple comparison was performed. *P < 0.05, **P < 0.01 and ***P < 0.001 vs no illumination group. ^##^*P* < 0.01 and ^###^*P* < 0.001 vs illuminated group. Data are shown as mean ± S.E.M. ONL: Outer Nuclear Layer, INL: Inner Nuclear Layer, GCL: Ganglion Cell Layer.

The effect of CD36 azapeptide ligand MPE-001 on subretinal inflammation was investigated by analysis of subretinal MP infiltration using IBA-1 staining of RPE flat mounts from blue light-challenged C57BL/6J wild type (WT, CD36^+/+^) and CD36^−/−^ mice (n = 6 and 3 mice/group respectively) (Fig. 1A,B). A significant increase in subretinal IBA-1+ cells was observed in the blue light-challenged CD36^+/+^ mice relative to their non-illuminated counterparts (Fig. 1A,C). IBA-1+ cell accumulation in the subretina was significantly decreased by 60% in blue light-challenged CD36^+/+^ mice treated with MPE-001 compared to vehicle-treated CD36^+/+^ mice (Fig. 1A,C). IBA-1+ cell accumulation in MPE-001 treated mice was equal to values observed in CD36^−/−^ mice (Fig. 1A–C). Furthermore, MPE-001 was ineffective in CD36^−/−^ mice, suggesting that subretinal inflammation induced by blue light exposure and mitigated by MPE-001 was CD36-dependent.

The relationship between subretinal inflammation and photoreceptor degeneration is well established^18–21^. The inhibitory effect of MPE-001 on MP infiltration into the subretinal space motivated further study of the capacity of the azapeptide CD36 ligand MPE-001 to preserve photoreceptor integrity in blue light-challenged mice. Glial fibrillary acidic protein (GFAP)^22,23^ is upregulated in response to MP activation, and can be used to index retinal degeneration. GFAP was mainly expressed in inner retinal layers in both CD36^+/+^ and CD36^−/−^ mice (Fig. 1D,E). Blue light exposure of WT mice caused photoreceptor degeneration evidenced by a thinner outer nuclear layer (ONL), a corresponding decrease in their function substantiated by lower a-wave and dependent b-wave electroretinographic amplitudes, as well as an increase in GFAP expression throughout the retina (Fig. 1D–M). MPE-001 administered to blue light-challenged WT mice preserved photoreceptor ONL thickness, restored a- and b-wave amplitudes, and reduced GFAP expression to values comparable to those seen in non-illuminated CD36^+/+^ mice. Hence, blue light-induced photoreceptor injury involved CD36.

Deficiency in the CX3C chemokine receptor 1 (CX3CR1) has been associated with subretinal MP accumulation with aging and exposure to light^18–20,24,25^. Employing CX3CR1-deficient mice (CX3CR1^−/−^) as a model of subretinal inflammation, exposure of CX3CR1^gfp/gfp^/CD36^+/+^ to blue light increased the abundance of IBA-1+ cells in the subretinal space and reduced ONL thickness relative to CX3CR1^gfp/gfp^/CD36^−/−^ mice illuminated with blue light (n = 3–4 mice/group), paralleling the effects of blue light on CD36^+/+^ and CD36^−/−^ mice (Fig. S1A,B). Treatment of blue light-exposed untreated CX3CR1^gfp/gfp^/CD36^+/+^ mice with MPE-001 reduced significantly (58%) subretinal MP and microglia infiltration, diminished GFAP expression and preserved ONL thickness (Fig. S1A,B). In contrast, the influence of blue light on GFAP expression and ONL degeneration were unaffected by MPE-001 treatment in CX3CR1^gfp/gfp^/CD36^−/−^ mice (Fig. S1B), illustrating further the role of CD36 in deleterious retinal inflammation and damage, and the pharmacologic efficacy of MPE-001.

### MPE-001 downregulates inflammatory markers of activated MPs and reduces photoreceptor degeneration *in vivo*

**Figure 2:**
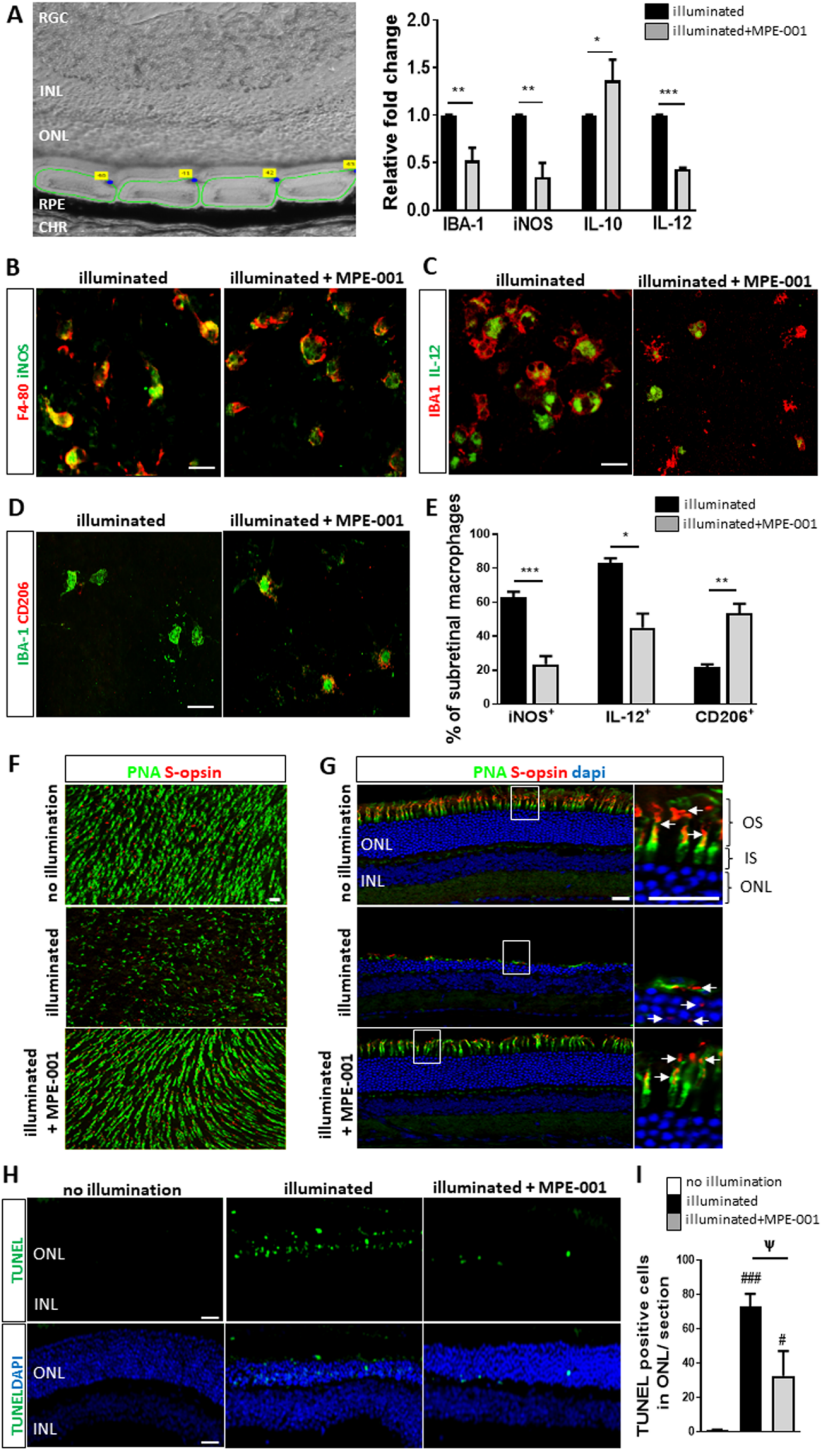
Azapeptide MPE-001 regulates inflammatory profile of subretinal MPs and reduces photoreceptor degeneration. (**A**–**H**) CD36^+/+^ mice were illuminated for 5 days with blue light. Subcutaneous injections of 289 nmol/kg MPE-001 were administered after 1 day illumination and pursued daily for 7 consecutive days. **(A)** Upper panel: area of retinal cryosections were microdissected between ONL and RPE and visualized with green circles. Lower panel: bar graphs of IBA-1, iNOS, IL-6, IL-10 and IL-12 mRNA expression levels in microdissected retinal cryosections. Cytokine analysis were normalized to 18 s rRNA. **(B)** Subretinal MPs stained for F4/80 (red) and iNOS (green) on RPE flat mounts as assessed by confocal microscopy. **(C)** Subretinal MPs stained with IBA-1 (red) and IL-12 (green) on RPE flat mounts as assessed by confocal microscopy. **(D)** Subretinal MPs stained with anti-IBA-1 (green) and anti-CD206 (red) antibodies on RPE flat mounts as assessed by confocal microscopy. Scale bar: 15 μm. **(E)** Percentage of subretinal MPs (IBA-1^+^ or F4/80^+^) expressing INOS, IL-12 or CD206. **(F)** Confocal microscopy of neuroretinal flat mounts (photoreceptors side) and **(G)** retina cryosections from illuminated CD36^+/+^ mice treated or not with MPE-001 stained with fluorescein PNA (green) and anti-S-opsin (red) antibody; nuclei were counterstained with dapi (blue). Magnifications of white square show length of cone outer segment with S-opsin distribution. Scale bar: 10 μm. **(H)** Retinal cryosections stained with TUNEL (green). Nuclear layers were stained with DAPI (blue). Scale bar: 25 μm. **(I)** Percentage of TUNEL^+^ cells in ONL cryosections of the retina. In (**A** and **E**) unpaired t-test was performed. *P < 0.05, **P < 0.01 and ***P < 0.001 vs illuminated group (n = 3-4 mice/group). In **I**, one-way ANOVA test with Newman-Keuls for multiple comparison was performed. ^#^*P* < 0.05 and ^###^*P* < 0.001 vs no illumination group. Ψ *P* < 0.05 vs illuminated group (n = 3-4 mice/group). Data are shown as mean ± S.E.M. RGC: Retinal Ganglion Cell. INL: Inner Nuclear Layer. ONL: Outer Nuclear Layer. RPE: Retinal Pigment Epithelium. CHR: Choroid.

Laser capture microdissection was used to determine mRNA levels of inflammatory markers in the area between the ONL and the RPE (Fig. 2A, area delineated by green line) in mice treated with MPE-001 compared with vehicle-treated control mice (n = 3–4 mice/group). MPE-001-treated mice exhibited significant reductions in the expression of iNOS, IL-12 and IBA-1 mRNA respectively by 65, 56 and 47%; whereas anti-inflammatory IL-10 mRNA was augmented by MPE-001 (Fig. 2A). Correspondingly, RPE flat mounts from animals treated with MPE-001 showed respectively 62% and 45% decreased numbers of iNOS^+^/F4/80^+^- and IL-12^+^/IBA-1^+^-stained cells compared to those from control mice (Fig. 2B,C,E). In addition, MPE-001 elicited increased expression of CD206, a surface marker of anti-inflammatory (M2-like) MPs (Fig. 2D,E). Consistent with a decrease in pro-inflammatory MPs and ensued preservation of the ONL structure (Fig. 1F)^26,27^, MPE-001 prevented against the degeneration of cone photoreceptor segments and the mislocalization of the S-opsin (Fig. 2F,G); it was also reducing apoptosis in the photoreceptors in WT mice exposed to blue light-illumination (Fig. 2H,I).

### MPE-001 diminishes TLR2-mediated proinflammatory cytokine and chemokine release *in vitro* and protects against photoreceptor degeneration

**Figure 3:**
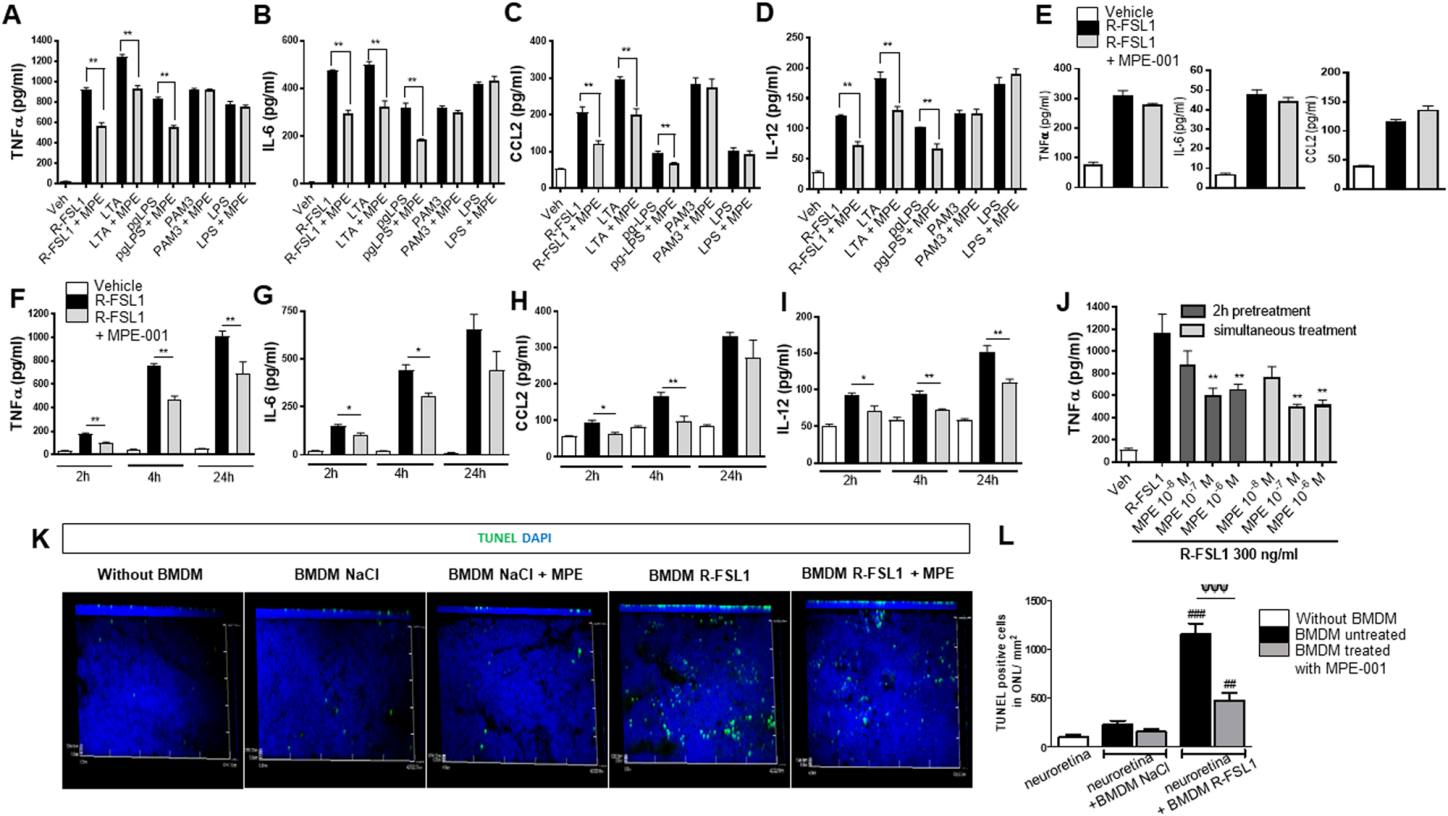
Selective inhibitory effect of CD36 ligand on TLR2-mediated pro-inflammatory cytokine secretion by MPs and ensued mitigation of photoreceptor apoptosis. (**A**–**D**) Pro-inflammatory cytokines TNFα, CCL2, IL-6 and IL-12 concentrations in supernatants of WT peritoneal MPs stimulated with selective TLR2/6 heterodimer agonist (300 ng/ml R-FSL1, 1 µg/ml LTA), TLR2/4 agonist (1 µg/ml *pg*LPS), TLR2/1 agonist (100 ng/ml PAM3CSK4) and TLR4 agonist (100 ng/ml LPS) for 4 h in the presence of 10^−7^ M MPE-001 or vehicle. (**E**) TNFα, IL-6 and CCL2 secretion in supernatants of peritoneal MPs from CD36^−/−^ mice treated with 10^−7^ M MPE-001 or vehicle, stimulation with 300 ng/ml R-FSL1 for 4 h. Data in **A–E** are representative of 5-6 independent experiments (n = 3-4/group). (**F**–**I**) Time-dependent release of TNFα, IL-6, CCL-2 and IL-12 secretion from WT peritoneal MPs treated or not with 10^−7^ M MPE-001 following stimulation with R-FSL1 for 2, 4 and 24 h (n = 4/group). (**J**) Dose-dependent inhibition of TNFα secretion in WT peritoneal MPs after 2 h pretreatment or simultaneous treatment with MPE-001 (10^−8^, 10^−7^ and 10^−6^ M) followed by 4 h stimulation with 300 ng/ml R-FSL1; (n = 8/group). (**K**) Confocal microscopy of flat mounts with z-stack projections of TUNEL (green) stained neuroretinal flat mounts incubated without or with BMDM stimulated with R-FSL1 or vehicle and treated or not with MPE-001. Nuclei are counterstained with DAPI (blue). (**L**) Numbers of TUNEL positive cells/mm^2^ in the ONL of neuroretinal explants incubated or not with monocytes in the different conditions (n = 3-4 eye/group). In (**A**–**I**) unpaired t-test was performed. *P < 0.05; **P < 0.01. In J and L one-way ANOVA test with Newman-Keuls for multiple comparison was performed. *P < 0.05; **P < 0.01. ^##^*P* < 0.01 and ^###^*P* < 0.001 vs neuroretina (without BMDM), ψψψ *P* < 0.001 vs BMDM untreated. Data are shown as mean ± S.E.M. RPE: Retinal Pigment Epithelium. CHR: Choroid.

Toll-like receptors (TLR) in association with cofactor proteins play crucial roles in innate immunity that trigger inflammatory responses^28^. The CD36, as co-receptor of TLR2/6 heterodimer, enhanced the TLR2-signaling pathway in the presence of its agonists, such as the diacylated lipoproteins LTA and R-FSL1^29–31^. Upon stimulation by specific ligands, the TLR2/6-CD36 complex triggers the activation of NFκB and MAPKs (P38 and JNK) which elicit an inflammatory response in MPs^13,29^. On the other hand, TLR2/1 heterodimer can be activated independently of the co-receptor CD36^29^. The role of CD36 in the mitigating effects of MPE-001 on TLR2-mediated inflammation was investigated in purified systemic MPs (peritoneal) from CD36^+/+^ and CD36^−/−^ mice, which were stimulated with IFNγ to induce a proinflammatory phenotype. The selectivity of MPE-001 to the CD36-TLR2 signaling pathway was demonstrated using a set of selective TLR agonists^29–31^: R-FSL1 and LTA for TLR2/6^32,33^, pgLPS for TLR2/4^34^, PAM3CSK4 for TLR2/1^35^, and LPS for TLR4^36^. Proinflammatory cytokines and chemokines were assayed by ELISA in the supernatant of WT macrophages after 4 h of stimulation by TLR agonists (n = 3–4/group). Increased secretion of tumor necrosis factor-α (TNFα), interleukin-6 (IL-6), C-C motif chemokine ligand 2 (CCL2) and IL-12 induced by R-FSL1, LTA and pgLPS was attenuated by MPE-001 (Fig. 3A–D, Table S1). MPs from CD36^−/−^ mice were less responsive to TLR2/6 stimulants and unresponsive to MPE-001 (Fig. 3E). MPE-001 was ineffective on inflammatory factor secretion elicited by PAM3CSK4 and LPS (Fig. 3A–D). The efficacy of MPE-001 on R-FSL1 inflammatory cytokine secretion in MPs from WT mice was time and dose-dependent (Fig. 3F–J). Similar effects of MPE-001 on R-FSL-1-induced cytokine secretion were also observed in human monocytes (Fig. S2A–C). Hence, upon its binding to the co-receptor CD36, MPE-001 decreased proinflammatory cytokines and chemokine release elicited by TLR2 specific agonists. These data showed for the first time that MPE-001 can modulate TLR2-mediated inflammation by acting on its co-receptor CD36.

Inflammation-associated photoreceptor loss is in part due to activated MPs (that co-express TLR2 and CD36) recruitment into the subretinal space^20,37–39^. To test this mechanism *ex vivo*, we induced a proinflammatory phenotype in ‘naïve’ murine bone marrow-derived MPs (BMDM) by stimulation with IFNγ and the TLR2 agonist R-FSL1. R-FSL1-stimulated and unstimulated BMDM were incubated facing the photoreceptor layers of neuroretinal explants (Fig. 3K). The extent of photoreceptor apoptosis was quantified by measuring TUNEL-positive cells per section (n = 3–4/group). Compared to photoreceptor layers exposed to unstimulated BMDM, photoreceptor layers exposed to R-FSL1-stimulated BMDM exhibited significant apoptosis (Fig. 3L), the latter of which was blocked by pretreatment of neuroretinal explants with MPE-001 (Fig. 3L).

### MPE-001 interfered with TLR2-signaling by disrupting TLR2-CD36 interaction

**Figure 4:**
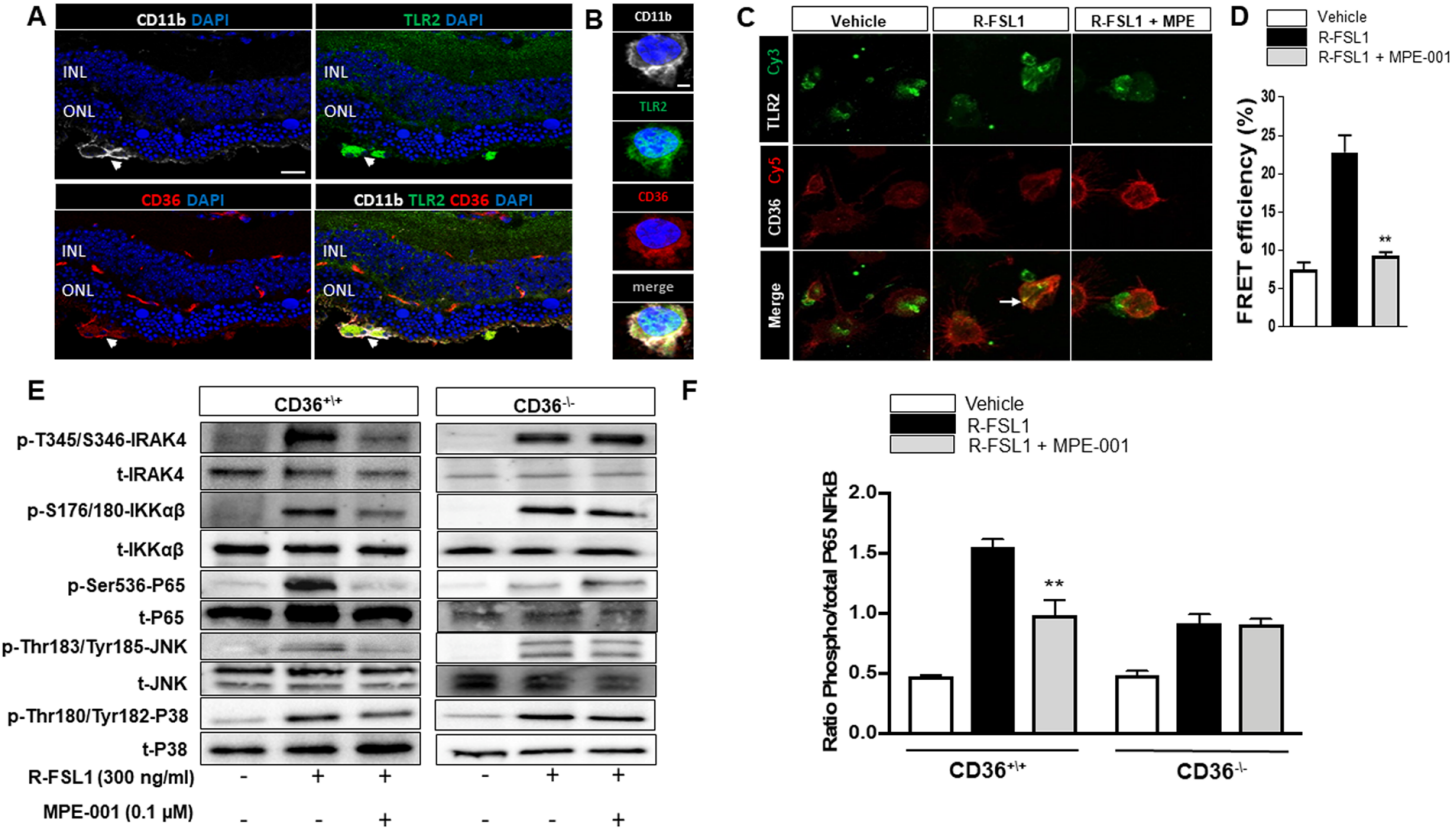
CD36 ligand disrupts TLR2-CD36 interaction modulated TLR2 heterodimer-signaling. (**A**) Confocal imaging of central retina cryosection stained with CD11b (white), CD36 (red), TLR2 (green), and DAPI (blue) from blue light-challenged WT mice. Scale bar = 25 μm. **(B)** High magnification (3X) shows subretinal CD11b-positive cells (white) with the co-localisation of CD36 (red) and TLR2 (green). Scale bar = 5 μm. **(C-F)** Peritoneal MPs were stimulated with 300 ng/ml R-FSL1 in the presence of 10^−7^ M MPE-001 or vehicle. **(C)** MPE-001 disrupted the interaction between CD36 labeled with Cy5 (red) and TLR2 labeled with Cy3 (green) as assessed by FRET after 5 min stimulation with R-FSL1. **(D)** Percentage of energy transfer measured using LSM-700 confocal microscope (Zeiss). Data in **B,C** are representative of 3-4 independent experiments. **(E)** Phosphorylated and total Western blot density bands of IRAK4, IKKαβ and P65-NFκB, JNK and P38 in peritoneal MPs from CD36^+/+^ and CD36^−/−^ mice stimulated with R-FSL1. **(F)** Quantification of P65-NFκB following stimulation of CD36^+/+^ and CD36^−/−^ peritoneal MPs with R-FSL1 using ELISA-based assay. Data in C-F are representative of 3 independent experiments (n = 3/group). In (**D**,**F**) one-way ANOVA test with Newman-Keuls post-test for multiple comparison was performed. *P < 0.05, **P < 0.01 and ***P < 0.001 vs R-FSL1. Data are shown as mean ± S.E.M.

Colocalisation of TLR2-CD36 was observed on subretinal MPs of blue light-exposed WT mice (Fig. 4A,B). Correspondingly, R-FSL1-induced colocalization of TLR2-CD36, and TLR2/TLR6 was also found in lipid rafts of peritoneal MPs in a MyD88-associated manner (Fig. S3D,E). Using FRET, we further explored the effects of MPE-001 on the CD36/TLR2 interaction. In both systemic (peritoneal) (Fig. 4C) and RAW MPs (Fig. S3A), TLR2 and CD36 were labeled respectively with the fluorescence donor Cy3 and acceptor Cy5. The effect of MPE-001 was studied by measuring energy transfer after photobleaching (n = 3–4 independent experiments). An increase of (fluorescent) energy transfer efficiency indicated a rapid association between CD36 and TLR2 on the membranes of both systemic and RAW MPS after stimulation with R-FSL1 (white arrow). MPE-001 attenuated R-FSL1-induced energy transfer efficiency between Cy3 and Cy5 in both types of MPs (Figs 4D and S3B), indicative of disrupted physical interaction between CD36 and TLR2. MPE-001-altered CD36/TLR2 interaction interfered with downstream signaling, as supported by decreased phosphorylation of IRAK4, IKKαβ, P65 NF-κB, JNK and p38 using Western blot (Figs 4E and S3C). The effect of MPE-001 on the key transcription factor P65 NF-κB has been validated by quantitative ELISA-based assay (Fig. 4F) (n = 3/group). However, MPE-001 had no effect on downstream signaling of CD36^−/−^ MPs stimulated with R-FSL1 (Figs 4E,F and S3C). CD36-dependent phagocytosis was also unaffected by MPE-001 (Fig. S4).

### MPE-001 decreases NLRP3 inflammasome

**Figure 5:**
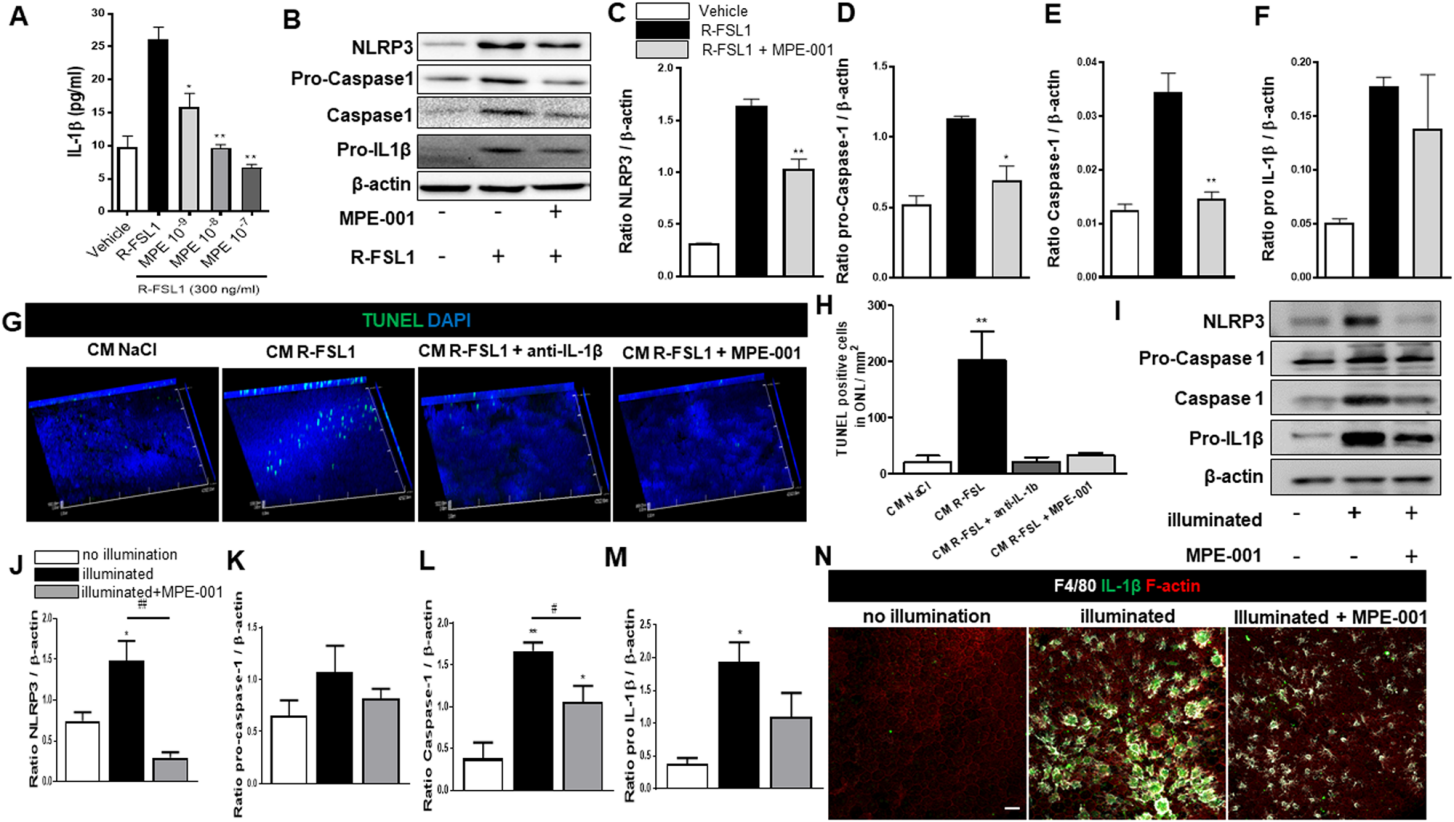
CD36 ligand downregulates inflammasome activation. (**A**–**F**) Peritoneal MPs were stimulated for 4 h with 300 ng/ml R-FSL1 and treated simultaneously with 10^−9^, 10^−8^ and 10^−7^ M MPE-001 or vehicle. 10 µM of ATP were added 30 min before the end of stimulation to induce IL-1β secretion. **(A)** IL-1β levels in supernatants of stimulated and MPE-001-treated peritoneal MPs (n = 4/group). **(B)** Western blot band density of inflammasome protein components in stimulated and treated peritoneal MPs. **(C**–**F)** Protein expression ratios of NLRP3, pro-Caspase1, Caspase 1 and pro-IL-1β to β-actin. Data are representative of 3 independent experiments (n = 3/group). **(G)** Representative neuroretinal flat mounts with z-stack projections of TUNEL (green) of CD36^+/+^ mice (n = 3 mice/group) observed by confocal microscopy. Neuroretina explants were incubated with conditioned media (CM) from BMDM or vehicle. Nuclei were counterstained with DAPI (blue). **(H)** TUNEL positive cells/mm^2^ in the ONL of neuroretinal explants incubated or not with CM from BMDM. **(I**–**N)** CD36^+/+^ mice (n = 3 mice/group) were illuminated for 5 days with blue light and subcutaneously injected with 289 nmol/kg per day of MPE-001 from 24 h after blue light exposure for 7 consecutive days. **(I)** Western blot band density of inflammasome proteins in retina from WT mice. Image representative of 3 independent experiments. **(J**–**M)** Expression ratios of NLRP3, pro-Caspase 1, Caspase 1 and pro-IL-1β/ β-actin in retina from CD36^+/+^ mice. **(N)** Confocal microscopy of RPE flat mounts from illuminated CD36^+/+^ mice stained with F4/80 (grey) and IL-1β (green); representative image of n = 3-4/group. F‐actin of RPE cells was counterstained with rhodamine phalloidin (red). Scale: 30 μm. In **A**,**C**–**M** one-way ANOVA test with Newman-Keuls for multiple comparison was performed. *P < 0.05 and **P < 0.01 vs R-FSL1 or vs CM NaCl. ^##^*P* < 0.01 and ^#^*P* < 0.05 vs no illumination. Data are shown as mean ± S.E.M.

The intracellular nucleation of CD36 ligands has been reported to trigger inflammasome activation^40^. We investigated next the ability of MPE-001 to mitigate CD36-dependent TLR2/6 stimulation of the inflammasome pathway. In IFNγ-primed peritoneal macrophages, induced IL-1β secretion on stimulation of TLR2/6 with R-FSL1 (n = 3–4/group) was associated with increased expression of NLRP3, pro-caspase1 and caspase1, all of which were attenuated by MPE-001 (Fig. 5A–F, Table S2), underscoring the critical photoreceptor cytotoxic effects of IL-1β as reported^41^. The role of inflammasome-generated IL-1β in inducing photoreceptor cytotoxicity was again studied on retinal explants incubated with conditioned media of R-FSL1-stimulated and unstimulated BMDM (n = 3/group) (Fig. 5G). Conditioned media from these R-FSL1-stimulated MPs induced an increase in apoptotic cell number in the photoreceptor layer, which was attenuated by anti-IL-1β antibody as well as by MPE-001 (Fig. 5G,H).

The effect of the CD36 modulator MPE-001 on the NLRP3-inflammasome was further studied *in vivo* on WT mice exposed to blue light (n = 3/group). MPE-001 suppressed blue light-induced increased expression of NLRP3, cleaved-caspase-1 and pro-IL-1β (Fig. 5I–M). Confocal microscopic analysis indicated colocalization of sub-retinal F4/80^+^ MPs, caspase-1 and IL-1β in illuminated tissues (Fig. 5N, Suppl. Fig. S5). MP activation was attenuated by MPE-001 (Fig. 5N), consistent with its other anti-inflammatory effects (Figs 1–3).

### Aerobic metabolic shift by MPE-001 contributes to attenuate pro-inflammatory MP activation

**Figure 6:**
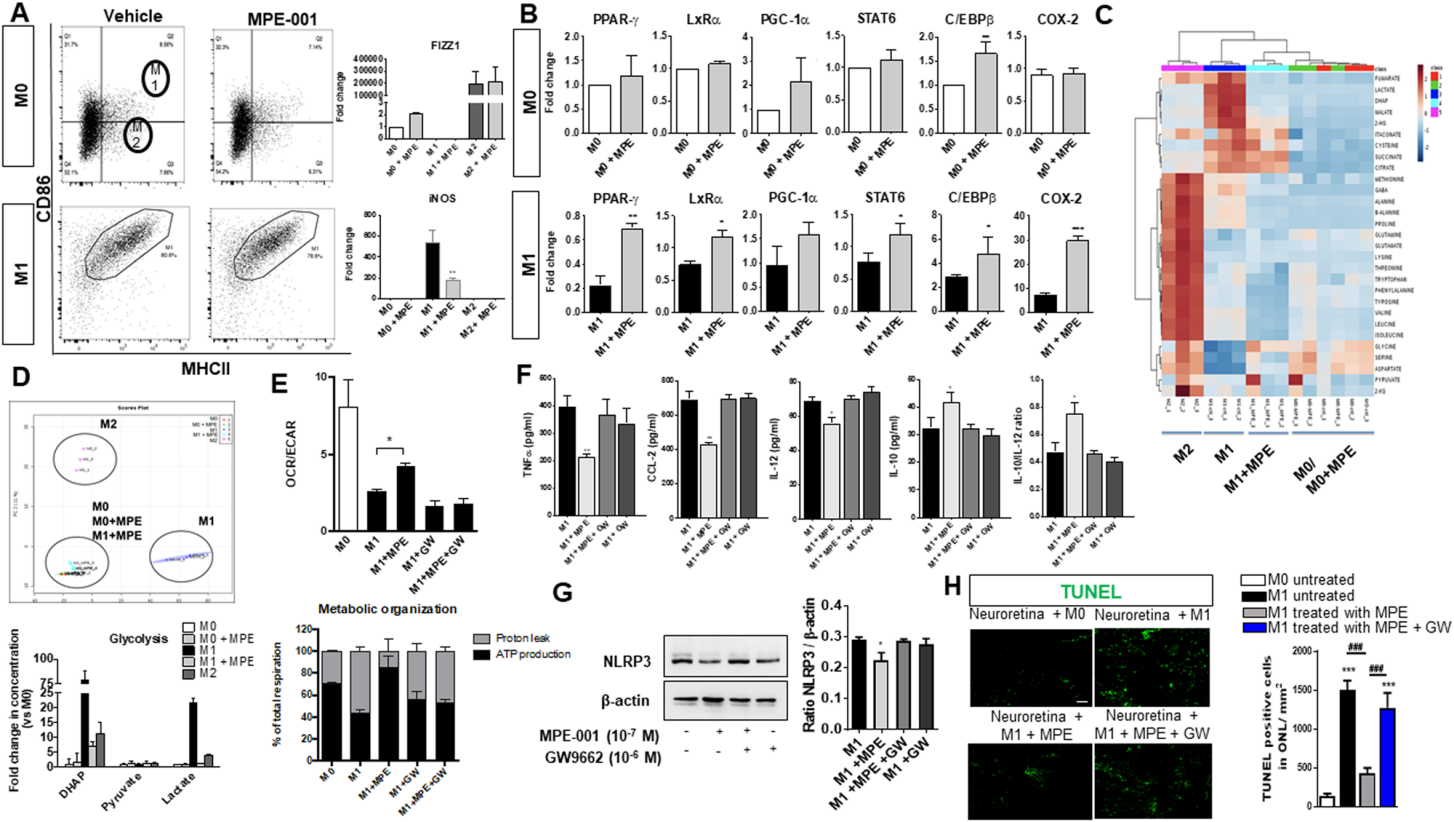
MPE-001 elicits a metabolic shift in M1 MPs. (**A**–**F**) BMDM were induced for 48 h to M0, M1 or M2 phenotype. BMDM were washed and treated 24 h with 10^−7^ M MPE-001 or vehicle. **(A)** Phenotypic analysis by flow cytometry of BMDM (M0 and M1) treated or not with MPE-001 using MHCII and CD86 and gene expression quantification of FIZZ1, YM-1 and iNOS. **(B)** Gene expression in M0 and M1 BMDM treated or not with MPE-001. **(C**–**E)** Metabolomic analysis. Heat map **(C)** and PCA score plot **(D)** (upper) of 29 metabolites extracted from M0, M1, and M2 BMDM and analyzed by GC-MS. M0 and M1 MPs were either treated with vehicle or MPE-001 (10^−7^ M) for 24 h. In PCA score plot, each point represents an independent MP subtype derived from primary cultures. 95% confidence regions are delimited by colored shapes. Lower: steady state levels of dihydroxyacetone phosphate (DHAP), pyruvate and lactate in M0, M1, and M2 MPs and analyzed by GC-MS. Data are representative of n = 3/group. (**E**) OCR/ECAR ratio (upper) and % of total respiration of M0 and M1 BMDM treated or not with MPE-001 (10^−7^ M) and PPAR-γ inhibitor GW9662 (10^−6^ M) for 24 h. Data are representative of 3 independent experiments (n = 3-5/group). **(F)** TNFα, CCL2, IL-12 and IL-10 secretion from M1 BMDM treated or not with MPE-001 (10^−7^ M) and PPAR-γ inhibitor GW9662 (10^−6^ M) for 24 h; n = 3-4/group. **(G)** NLRP3 expression in M1 BMDM treated or not with MPE-001 and PPAR-γ inhibitor GW9662 (10^−6^ M) for 24 h. Expression ratio of NLRP3/ β-actin band intensities are presented in histogram (n = 4/group). **(H)** Confocal microscopy of TUNEL (green) stained neuroretinal flat mounts (photoreceptors side) incubated with BMDM stimulated with R-FSL1 (300 ng/ml) or NaCl and treated or not with MPE-001 (10^−7^ M) and PPAR-γ inhibitor (10^−6^ M) (n = 3/group). Numbers of TUNEL positive cells/mm^2^ in the ONL of neuroretinal explants incubated or not with macrophages in the different conditions. In **A** unpaired t-test was performed. *P < 0.05; **P < 0.01. In **B**,**E**,**F**,**G** and **H** one-way ANOVA test with Newman-Keuls for multiple comparison was performed. *P < 0.05; **P < 0.01; ***P < 0.001. ^###^P < 0.001 vs M1 treated with MPE-001. n = 4-6 eyes/group, data are shown as mean ± S.E.M. Scale bar: 100 µm.

To determine if the anti-inflammatory CD36-dependent mechanism of action of MPE-001 influenced MP polarization, experiments were performed employing undifferentiated and differentiated BMDM (n = 3–5/group). BMDM were polarized into classically activated pro-inflammatory (M1-differentiated BMDM) and anti-inflammatory M2 phenotype^42^. MPE-001 did not alter the expression of either the M1 marker CD86 in M0 and M1-differentiated BMDM, nor that of the M2 marker FIZZ1 in the same cell types (Fig. 6A); similar observations were made using peritoneal MPs (Fig. S5A). On the other hand, pro-inflammatory factors, notably iNOS, TNFα, CCL-2, and IL-12, were attenuated by MPE-001 in M1-like differentiated BMDM (Fig. 6A,F), consistent with observations in murine peritoneal macrophages (Fig. 3A–D) and human monocytes (Fig. S2). Considering MPE-001 diminished expression of pro-inflammatory factors and altered the expression of some MP polarization markers, such as CD206 (Fig. 2D,E), we interrogated whether CD36 could affect MP activity by altering other processes such as metabolism. Notably, CD36 can dampen MP migration^43,44^, and associated inflammatory responses by inducing expression/activation of peroxisome-proliferator-activated receptor-γ (PPAR-γ)^45,46^, which in turn augments β-oxidation^47^. Treatment with MPE-001 caused M1-differentiated BMDM (but not M0, nor M2-differentiated BMDM) to display an overall increase in *PPAR-γ*, liver X receptor-α (*LxRα*), peroxisome proliferator-activated receptor gamma coactivator 1-α (*PGC-1α*), signal transducer and activator of transcription-6 (*STAT-6*), CCAAT-enhancer-binding proteins β (*C/EBPβ*) and cyclooxygenase-2 (*COX-2*) gene expression (Fig. 6B, gene array presented in Fig. S5D). This induction of PPAR-γ/PGC-1α, master regulators of mitochondrial biogenesis and function, suggested that MPE-001 could promote oxidative metabolism in MPs. To gain further insights into metabolic changes induced by MPE-001, we studied by GC-MS 29 metabolites extracted from different MP subtypes. Heat map analysis of the metabolites revealed strong differences between M1 and M2 subtypes and a strong shift from M1 to M0-like subtype upon treatment with MPE-001 (Fig. 6C). Indeed, unsupervised PCA analyses using two principal components revealed that, except for control M0 and M0 treated with MPE-001, each MP subtype formed a clearly distinct and non-overlapping group (n = 3/group). Although azapeptide MPE-001 had no effect on M0-subtype MPs metabolism (Fig. 6D), MPE-001 affected profoundly the metabolite profile of M1-subtype MPs, as these cells exhibited a pattern distinct from (untreated) M1-subtype and M2-subtype MPs, suggesting that MPE-001 treatment caused M1-subtype MPs to switch to a M0-like subtype, consistent with the shift in bioenergetic metabolism as reflected by the changes in mitochondrial respiration (Fig. 6E). Treatment of M1-differentiated BMDM with MPE-001 caused a reduction in glycolysis, indicated by markedly diminished lactate and dihydroxyacetone phosphate (DHAP) concentrations (n = 3/group) (Fig. 6D). This finding was further validated by bioenergetic assay showing that MPE-001 treatment of M1-differentiated BMDM induced a significant increase of OCR/ECAR ratio, an indicator of mitochondrial oxygen consumption rates (OCR) and extracellular acidification rates (ECAR) (Fig. 6E), suggesting a decrease in glycolysis and an increase in mitochondrial oxidative phosphorylation (n = 3–5/group). Indeed, M1-subtype MPs treated with MPE-001 nearly doubled respiration in support of ATP synthesis compared to untreated M1-subtype MPs which are strongly uncoupled (Fig. 6E). Once again, we confirmed these observations in peritoneal MPs, as MPE-001 increased expression of PPAR-γ, PGC-1α and oxidative phosphorylation enzymes, indicating its effect on bioenergetic reprogramming in ATP production in M1-differentiated BMDM (Fig. S5B,C). Inhibition of PPAR-γ using the selective inhibitor GW9662 was performed to assess the critical role of MPE-001 modulation of PPAR-γ/PGC-1α (along with LxRα) on MP bioenergetics^47^, as well as on the inflammatory profile^45,46^. PPAR-γ inhibition abrogated MPE-001-induced increase in OCR/ECAR ratio in M1-differentiated BMDM (Fig. 6E) and blocked the corresponding anti-inflammatory actions including suppression of NLRP3 expression by MPE-001 (n = 3–4/group) (Fig. 6F,G). The combined effects of PPAR-γ inhibition negated the anti-apoptotic activity of MPE-001 on photoreceptors (n = 3/group) (Fig. 6H). Collectively, the findings illustrate that CD36 modulation by the azapeptide MPE-001 causes both interference of TLR2-mediated inflammation as well as alters MP metabolism which in turn regulates the activity of the common NLRP3 pathway.

### Subretinal MPs in inflammatory retinal disease of elderly subjects display similar profiles of CD36 and TLR2 expression to those observed in light-induced inflammation in mice

Human retina from healthy elderly donors and from patients presenting age-related retinal inflammation were stained with IBA-1. Their subretinal myeloid cells were examined in RPE flat mounts (counter staining with rhodamine phalloidin, Fig. S6B,C) and compared to retina from blue light-illuminated mice (Fig. S6A). The immune cell accumulation that was found in retinas of elderly human donors with inflammatory eye disease was comparable to that observed in mice exposed to blue light irradiation (Fig. S6A,C). RPE flat mounts showed expression of CD36 (Fig. S6D) and TLR2 (Fig. S6E) in all subretinal IBA-1^+^ MPs. Atrophy of the RPE correlated with the accumulation of IBA-1^+^ MPs (Fig. S6C magnification) and contrasted with the regular shape of RPE cells from an elderly donor without subretinal inflammation (Fig. S6B magnification).

## Discussion

**Figure 7:**
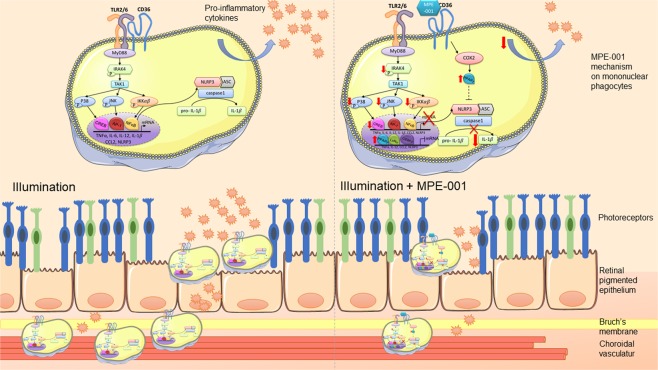
Schematic representation of major mechanisms involved in immuno-metabolic modulation of retinal inflammation by CD36 ligand MPE-001. Retinal inflammation induced by photo-oxidative stress induces subretinal MP accumulation, pro-inflammatory cytokine production and photoreceptor degeneration (left panel). Treatment with MPE-001 diminishes subretinal MP accumulation, reduces pro-inflammatory cytokine production and as a result protects photoreceptors (right panel). MPE-001 exerts these benefits by dissociating the CD36-TLR2/6 complex, attenuating NF-κB and NLRP3-dependent inflammasome activation, and in addition by increasing COX-2 and PPAR-γ which also mitigates inflammation as it induces an aerobic metabolic shift in MPs (right panel).

Inflammatory processes play critical roles in the pathogenesis of various retinal diseases^48^. Although regulation of the complement system has garnered significant contemporary attention for the treatment of degenerative retinal diseases, such as age-related macular degeneration^49^, alternative strategies to curb chronic inflammation driven by MPs merit further study to address underlying causes of tissue damage. Neuronal cell death, such as that seen for photoreceptors and retinal ganglion cells, is often the consequence of MP activation through TLR pattern recognition and intracellular signaling to stimulate genes encoding pro-inflammatory cytokines^9^. TLR2 pro-inflammatory function has been postulated to contribute to RPE apoptosis due to inflammation and oxidant stress^8^. Considering their important roles in degenerative retinal disease pathology, TLR signaling pathways have emerged as promising targets for mitigating MP-driven inflammation; however, direct TLR inhibition and antagonism have to date had limited success in the clinical setting^50,51^. Alternatively, cofactor proteins which associate with TLRs and modulate their activity, represent an unexplored means for disrupting their signaling. Studying modulators of CD36, we have identified the azapeptide ligand MPE-001 that binds this cofactor protein and consequently interferes with TLR2 signaling. Herein, we provide mechanistic insights into the mode of action of MPE-001. After binding to the hydrophobic region of CD36^52,53^, MPE-001 disrupts the interaction between CD36 and the TLR2/6 heterodimer at the MP cell membrane, and subsequently perturbs downstream signaling by attenuating the relevant photo-oxidative stress-triggered pro-inflammatory cascade. Concomitantly, MPE-001 enhances the metabolic rate of MPs through PPAR-γ induction which in turn contributes to suppression of inflammation via a common NLRP3 link with the TLR2 pathway; abrogation of inflammation preserves photoreceptor integrity (see schematic diagram in Fig. 7).

The class B scavenger receptor CD36 was first identified as a fatty acid transporter involved in energy metabolism^54^, and later implicated in TLR-dependent inflammatory response and sterile inflammation featuring inflammasome activation in MPs^40^. The selective azapeptide CD36 ligand MPE-001 has now been shown to modulate activation of the TLR2/6 heterodimer and downstream signaling in MPs, resulting in decreased pro-inflammatory cytokine and chemokine release, and mitigation of the influx, activation and accumulation of MPs into the subretinal space, which is normally devoid of immune cells^24^. The consequences of MPE-001 treatment include attenuation of inflammation which causes RPE and photoreceptor layer degeneration^55^, as demonstrated in blue light-exposed mice *in vivo*. The efficacy of MPE-001 was further substantiated in the CX3CR1-deficient murine model in which the absence of CX3CR1 accelerates tissue damage and retinal degeneration due to increased presence of mononuclear phagocytes in the retina upon exposure to photo-oxidative injury^20^. By reducing proinflammatory cytokine levels and mononuclear phagocyte recruitment, MPE-001 exhibited cytoprotective effect, prevented photoreceptor loss, and preserved significantly retinal function after exposure to conditions of photo-oxidative stress that mimic chronic inflammation^56^. Moreover, MPE-001 modulated the assembly of cytoplasmic components of the inflammasome and decreased IL-1β release in subretinal MPs. Although CD36 has been shown to act as non-opsonic phagocytic receptor^57^, and to cooperate with TLR4 in bacterial endocytosis and phagocytosis by MPs^58^, MPE-001 interfered selectively with TLR2 stimulation without altering the MP phagocytic function. The manner by which MPE-001 preserved the innate immune response is suggestive of selective biased allosteric modulation of the CD36 interaction with TLR2; these combined observations were consistent in MPs from distinct sources, including in human monocytes.

The role of the CD36-TLR2 interaction in mediating inflammation and ensuing neurotoxicity offers a novel target for therapeutic intervention. The prototype MPE-001 disrupted association between CD36 and TLR2 proteins labeled with fluorescent probes as demonstrated by an observed reduction of the energy transfer caused on binding to the TLR2 agonist R-FSL-1. Consequently, the normal TLR2-signaling pathway was interrupted by MPE-001 as demonstrated by diminished phosphorylation of various downstream signals of TLR2^59,60^.

Cell-specific responses may be mediated by CD36, which interacts with multiple ligands and binding partners: e.g., TLR heterodimers, β1 and β2 integrins^61^, and tetraspanins^62^ to activate NF-κB, NLRP3, Src/Lyn/Fyn, MAPKs and TGFβ signalling pathways. Among multiple CD36 lipid-related ligands, oxidized phospholipids were shown to promote the activation of TLR4/6-dependent innate immune response^63,64^. Binding of oxidized LDL is associated with the upregulation of inflammatory cytokine expression and inflammasome stimulation to trigger pro IL-1β and NLRP3 activation^40^. On binding to CD36, MPE-001 decreased IL-1β release through a modulatory effect on the activation of the TLR2/6-CD36 complex by ligands such as the diacylpeptide agonist R-FSL1. The modulatory roles of MPE-001 on TLR2-dependent inflammatory processes and sterile inflammation, are both mediated through NLRP3 activation. As a co-receptor of TLR2, CD36 activates AP-1 and triggers gene transcription of proinflammatory cytokines, primarily through activation of c-Jun N-terminal kinase (JNK) and P38^58^. Our results indicate that azapeptide MPE-001 decreased AP-1 activation by reducing phosphorylation of JNK and P38 in activated MPs.

MPs in the subretinal space and many other tissues are characterized as resident and invading pathology-triggered inflammatory-types. Analogous to the adaptive immune system in which Th1 and Th2 cells have been characterized, MPs have also been subdivided based on their cytokine production^42^. MPs activated by DAMPs are pro-inflammatory, anti-angiogenic and potentially neurotoxic. Those stimulated by anti-inflammatory cytokines (e.g. IL-4) display pro-angiogenic properties, promote phagocytosis and are anti-inflammatory. However, the spectrum of MP phenotypes is broader than traditionally specifically-labelled M1 and M2 subtypes^65,66^. The inflammatory profile of MPs is also affected by metabolic rate, such that inhibition of glycolysis or oxidative phosphorylation alters respectively M1 or M2 activation^67,68^. CD36 affects metabolic pathways. CD36 modulators enhance influx of oxidized LDL and separately, efflux of cholesterol via ABCA1/G1 transporters through a PPAR-γ-dependent process^69^. PPAR-γ repression (antagonism) enhances the glycolytic metabolic pathway^70,71^, stimulates oxidative phosphorylation^72^, and augments concomitantly anti-inflammatory cytokines to curtail the pro-inflammatory actions of excessive CD36 induction^73^. Our findings *in vitro* and *in vivo* concur with these concepts, which reveal that CD36 azapeptide ligands exert anti-inflammatory properties by affecting the TLR2-inflammasome pathway and by shifting metabolic rate to increase oxygen consumption by influencing the PPAR-γ pathway. Accordingly, MPE-001 acts by inhibiting the signaling of CD36 to certain pathways (specifically NF-κB-inflammasome) and activating others (notably PPAR-γ-PGC1α), consistent with the biased signaling actions that we have reported for other CD36 ligands^16,69^.

The effects of MPE-001 in decreasing inflammation and IL-1β release may go beyond MPs^74^, and extend to other cells expressing CD36 and inflammasome components, such as the RPE and choroidal endothelium^75^. Notably, in the late stages of human RPE degeneration, NLRP3-inflammasome activation and increased levels of IL-1β correlate with oxidative stress that leads to lipid peroxidation end products such as 4-hydroxynonenal and carboxyethylpyrrole^75^. Considering IL-1β release elicits subretinal accumulation of MPs responsible for cone segment degeneration with loss of high visual acuity^21^, the potential for MPE-001 to reduce IL-1β release may be exploited to prevent cone cell loss in geographic atrophy^21^, consistent with observations made herein. In addition, downregulation of expression of pro-inflammatory inducible nitric oxide synthase and IL-12 in subretinal MPs on treatment with MPE-001 *in vivo* was also accompanied by increase in expression of anti-inflammatory IL-10 which may further dampen NLRP3 expression, inflammasome assembly and caspase-8 activation^76^.

In summary, we have shown for the first time that MPE-001, a selective azapeptide ligand of CD36, can specifically modulate the CD36-TLR2 interaction and the induction of PPAR-γ/PGC-1α. Consequently, MPE-001 mitigated inflammation and ensuing neurotoxicity, consequences that are regularly observed in degenerative outer- and sub-retinal disorders. Considering subretinal inflammation with accumulation of activated MPs is prevalent in retina from elderly human patients and mirrored in mice subjected to photo-oxidative stress, modulators such as MPE-001 offer promise as a novel prototype for therapeutic targeting of the CD36 receptor to mitigate chronic MP-driven inflammation in vision-threatening maladies, such as retinitis pigmentosa, diabetic retinopathy and age-related macular degeneration.

## Materials and Methods

### Antibodies and reagents

Antibodies against phospho-P65 (3033), phospho-JNK (4668), phospho-P38 (9211), phospho-IRAK4 (11927), phospho-IKKαβ (2697), total JNK (9252), P38 (9212), total IRAK4 (4363), total IKKβ (2678), TLR2 (13744), TLR6 (12717) and MyD88 (4283) were purchased from Cell Signaling Technology (Danvers, MA, USA). Antibodies against total P65 (sc-8008) were from Santa Cruz Biotechnology (Dallas, TX, USA). Antibodies against APC/Cy7 anti-mouse F4/80 (123117) and PerCP/Cy5.5 anti-mouse CD80 (104721) were purchased from Biolegend (San Diego, CA, USA). Antibodies against CD36 (NB400-145) were from Novus Biologicals (Littleton, CO, USA). Antibodies against Flottilin1 (ab41927), IL-1β (ab9722), CD36 (ab800080), CD36 (ab125116), F4/80 (ab6640), Total OXPHOS Rodent Cocktail (ab110413), PGC-1α (ab54481) and PPAR-γ (ab209350) were from Abcam (Cambridge, United Kingdom). Antibodies against TLR2 (mab-mtlr2) were from Invivogen (San Diego, CA, USA). Antibodies against TLR2 (orb191498) was from Biorbyt (Cambridge, United Kingdom). Antibodies against IBA-1 (019-19741) were from Wako (Neuss, Germany). Antibodies against PE-IL-12 (C17.8) were from eBiosciences (San Diego, CA). Anti-GFAP (Z0334) was from Dako Agilent (Santa Clara, CA, USA). Rhodamine phalloidin (00027) was from Biotium (Scarborough, Ontario, Canada). Fluorescein peanut agglutinin (PNA) (FL-1071) was from Vector Laboratories (Burlingame, CA, USA). Antibodies against Opsin-blue (AB5047) from Millipore Sigma (Oakville, ON, CA). AlexaFluor 488-, 594-conjugated secondary antibodies were from Invitrogen (Carlsbad, CA, USA). AlexaFluor 647-conjugated secondary antibodies were from New England Biolabs (Ipswich, CA, USA). 4′,6-diamidino-2-phenylindole (DAPI) and GW9662 (M6191) were from Sigma-Aldrich (Saint-Louis, MO, USA). Antibodies against iNOS (PA3-030A) and West Femto Chemiluminescent Substrate (PI-34095) were from Thermo Scientific (Waltham, MA). Horse radish peroxidase (HRP)-conjugated secondary goat anti-rabbit IgG was from Jackson Immunoresearch, West Grove, PA, USA). rmIFNγ (575306), rmM-CSF (576406), APC/Cy7 anti-mouse F4/80 (123117), Brilliant Violet 421 anti-mouse I-A/I-E (107631), PE/Cy5 anti-mouse CD86 (105015), PE anti-mouse CD206 (141705) and TruStain fcX (anti-mouse CD16/32) (101319) were from Biolegend (San Diego, CA, USA). Energy donor Cy3 (ab188287) and energy acceptor Cy5 (ab188288) were from Abcam (Cambridge, United Kingdom). Fibroblast-stimulating lipopeptide (R-FSL-1) was from EMC microcollections GmbH (Tübingen, Germany). Lipoteichoic acid (LTA) from S. aureus, Pam3CysSerLys4 (Pam3Csk4) and Porphyromonas gingivalis (pgLPS) were from Invivogen. Lipopolysaccharide (LPS) from *E.Coli* (0111:B4) and ATP (FLAAS-1VL) were from Sigma-Aldrich (Oakville, ON, Canada). Monocyte Isolation Kit (130-100-629) was from Miltenyi Biotec (Auburn, CA, USA). ELISA kits against TNFα (88-7324), IL-6 (88-7391), CCL2 (88-7391), IL-12 (88-7121) and IL-1β (88-7013) were from eBiosciences (San Diego, CA, USA). Total/Phospho InstantOne™ ELISA against NFκB p65/RelA (85-86083) was from eBiosciences (San Diego, CA, USA). Cocktail tablets of protease and phosphatase inhibitors (PI88666, PI88667) and bicinchoninic acid protein assay (PI23223, PI23224) were from Pierce Biotechnology (Waltham, MA, USA). Murine IL-4 (214-14) and IL-13 (210-13) were from Peprotech Inc. (Rocky Hill, NJ, USA).

### Human eyes

Eyes from aged humans (77 and 79 years old) were obtained from the Eye Bank of Canada. The human clinical protocol and informed consent forms were approved by the CHU Sainte-Justine ethics committee and adhered to the tenets of the Declaration of Helsinki. The eyes were fixed for 4 h with 4% paraformaldehyde (PFA) and dissected in a petri dish containing Hank’s Balanced Salt Solution HBSS (Invitrogen). The posterior part of the eye (cornea, lens) were removed and the anterior part was cut in 5 pieces and prepared for flat mounts.

### Animals

*Cx3cr1*^GFP/GFP^ and *CD36*^−/−^*Cx3cr1*^GFP/GFP^ mouse strains on *C57Bl/6J* background were a generous gift of Dr. Florian Sennlaub (Institut de la vision, INSERM, Paris, France)^77^. *CD36*^−/−^ mice and their littermate (CD36^+/+^) controls were generated as previously described^78^. Mice were housed and maintained at local animal facilities under a 12 h:12 h light/dark cycle unless otherwise indicated.

### Blue light illumination model

Three to four-month-old mice were exposed to blue LED-light (Yescom USA, Inc.) for 5 days at an illuminance of 6000 lux without previous dark-adaptation. For pupil dilatation, ophthalmic atropine solution 1% (Alcon) was applied to both eyes daily. MPE-001 (289 nmol/kg) was administered *s.c*. at 24 h following blue light exposure for 7 consecutive days. At the end of the blue light exposure period, the mice were maintained on a 12:12 h light: dark cycle for 3 days before being sacrificed.

### Isolation and culture of mouse peritoneal macrophages

Unstimulated peritoneal macrophages were harvested by washing the peritoneal cavity of 12-week-old *C57BL/6J* and *CD36*^−/−^ male mice using 10 mL Dulbecco’s Modified Eagle’s Medium (DMEM) cell-culture medium. Peritoneal macrophages were purified by depletion of non-target cells using the Monocyte Isolation Kit (Miltenyi, 130-100-629) according to the manufacturer’s instructions. Flow cytometry analysis indicated that the cell population contained above 98% F4/80^+^CD80^+^ cells. Purified peritoneal macrophages were plated in DMEM containing 10% Fetal Bovine Serum (FBS) and 20 ng/mL interferon γ (IFNγ) at 37 °C in a 5% CO_2_-enriched atmosphere. After 48 h, cells were washed twice with PBS to remove IFNγ and FBS. Peritoneal macrophages were then weaned off FBS by incubation for 2 h with DMEM containing 0.2% Bovine Serum Albumin (BSA) prior to stimulation.

### Bone marrow-derived MPs (BMDM)

BMDM were isolated from femurs and tibias of 8-12-week-old C57BL/6 mice. Bones were cut in half and put into 0.6 mL pierced micro-centrifuge tubes. The tubes were put into 2 mL micro-centrifuge tubes containing 200 μl DMEM and centrifuged at 6000 g for 2 min. Bone marrows were suspended in DMEM/10% FBS, and cells were cultured for 7 days in the presence of macrophage colony-stimulating factor (mrM-CSF; 40 ng/mL) and cultured for 7 days. Flow cytometry analysis indicated that the cell population contained above 98% F4/80^+^CD80^+^ cells. BMDM were then stimulated for 48 h with IL-4 and IL-13 (20 ng/mL) for M2 differentiation, or with R-FSL1 (300 ng/mL) and IFNγ (20 ng/mL) for M1 differentiation. M0 (unstimulated cells), M1 or M2 BMDM were treated or not for 24 h with 10^−7^ M MPE-001 and/or with 10^−6^ M PPAR-γ inhibitor (GW9662).

### Cytokines and intracellular signaling molecules activation assays by ELISA

Peritoneal macrophages (2.5 × 10^5^) purified from WT or CD36^−/−^ mice were seeded on 48-well plates. Peritoneal macrophages were exposed to azapeptide MPE-001 (10^−8^, 10^−7^, 10^−6^ M) or vehicle for 0 or 2 h, then stimulated with either 300 ng/mL R-FSL1, 1 µg/mL LTA (TLR2/6 selective agonists), 100 ng/mL Pam3Csk4 (TLR2/1 selective agonist), 1 µg/mL pgLPS (TLR2/4 selective agonist) and 100 ng/mL LPS (TLR4/6 selective agonist). Cell supernatants were removed after 2, 4 or 24 h. TNFα, IL-6, CCL2 and IL-12 levels in cell supernatants were assayed with Ready-SET-GO ELISA kits (eBiosciences). All samples were measured in triplicate according to the manufacturer’s instructions. Signaling molecule phosphorylation of NFκB p65/RelA, JNK 1/2 and p38 MAPK were documented on cell lysates after 10, 30 or 360 min of stimulation with R-FSL1, respectively, using InstantOne™ ELISA (eBiosciences). All samples were measured in triplicate according to the manufacturer’s instructions.

### Western blotting

For *in-vitro analysis*, proteins were extracted from peritoneal macrophages (3 × 10^6^) seeded in 6-well plates and treated with azapeptide MPE-001 (10^−7^ M) or vehicle and stimulated with 300 ng/mL R-FSL1 for 5, 30 or 360 min. *For in-vivo analysis*, proteins were extracted from RPE/choroids from mice exposed or not to blue light and treated with or without MPE-001. Cells and tissues were washed with PBS then lysed for 30 min in ice-cold radioimmunoprecipitation assay (RIPA) buffer (150 mM NaCl, 50 mM Tris-HCl, 1% Triton X-100, 0.2% SDS, 50 mM NaF, 2 mM EDTA, pH = 7.4) containing protease and phosphatase inhibitors (Pierce Biotechnology). Cell and tissue lysates were centrifuged at 10,000 g for 30 min at 4 °C. The protein concentration of supernatants was determined by the BCA assay (Pierce Biotechnology). Equal amounts (30 μg) of protein extract were mixed with Laemmli buffer, heated for 5 min at 95 °C, separated on 7.5% SDS-polyacrylamide gel (SDS-PAGE) and transferred electrophoretically to polyvinylidene difluoride (PVDF) membranes (Bio-Rad Laboratories) for immunoblotting. Membranes were incubated for 1 h at room temperature in 150 mM NaCl and 10 mM Tris-HCl, 0.05% Tween 20, pH 7.6 (Tris-buffered saline Tween 20, TBST) containing 5% BSA and washed briefly in TBST followed by overnight incubation at 4 °C with primary antibodies (P65, phospho-P65, JNK1/2, phosphor-JNK1/2, P38, phospho-P38, IRAK4, phospho-IRAK4, IKKαβ, phospho-IKKαβ, NLRP3, Caspase-1, IL-1β or pro-IL-1β all used at 1:500 vol:vol). Antibodies against P65, JNK1/2, P38 and β-actin were used as internal controls. After the washing steps, blots were incubated for 1 h at room temperature with HRP-conjugated secondary antibodies diluted at 1:5000 vol:vol (Jackson Immunoresearch). Immunoblotted bands were detected by enhanced chemiluminescence (ECL) with West Femto chemiluminescent substrate (Thermo Scientific) using ChemiDoc MP Imaging System (Bio-Rad Laboratories). Image analysis was performed using ImageLab 5.2 software (Bio-Rad Laboratories).

### Colocalization of CD36-TLR2 in lipid rafts

Peritoneal macrophages (10^7^) plated in petri dishes (10 mm^2^) were stimulated for 5 min with the TLR2 agonist (R-FSL1, 300 ng/ml) or vehicle. Cells were lysed in 1 mL of RIPA buffer (150 mM NaCl, 50 mM Tris-HCl, 1% Triton X-100, 50 mM NaF, 2 mM EDTA, pH 7.4) containing protease and phosphatase inhibitors (Pierce Biotechnology) for 1 h on ice. Cell lysates were mixed with an equal volume of 5% sucrose in RIPA and placed at the bottom of a centrifuge tube then subjected to sucrose density gradient. Samples were overlaid with 1 ml of 40%, 1 mL of 30%, 2 mL of 25%, 2 mL of 20%, 2 mL of 15% and 2 mL of 10% sucrose in RIPA, then centrifuged at 100,000 g for 16 h at 4 °C. Fractions (1 mL) were gently removed from the top of the gradient and subjected to centrifugal filtration Microcon (Millipore, MRCPRT010) with a membrane Nominal Molecular Weight Limit (NMWL) of 10 kDa for protein concentration and desalting. N-octylglucoside (25 mM) was added to each fraction to solubilize lipid rafts and equivalent portions of each fraction were analyzed by SDS-PAGE and immunoblotted using primary antibodies: anti-CD36 (NB400-145), anti-TLR2, anti-MyD88, anti-Flottilin1 or anti-TLR6, all used at 1:500 vol:vol. After the washing steps, blots were incubated for 1 h at room temperature with HRP-conjugated secondary antibodies (1:5000 vol:vol, Jackson Immunoresearch). Bands were detected by ECL as described above.

### IL-1β and NLRP3 protein assays

Peritoneal macrophages (1.5 × 10^6^) were seeded in 12-well plates in DMEM/10% FBS overnight. Cells were weaned off FBS for 2 h with DMEM containing 0.2% BSA, then were treated with MPE-001 (10^−7^ M) or vehicle and stimulated with 300 ng/mL R-FSL1 for 4 h. Thirty minutes before the end of stimulation, 10 µM of ATP was added to the cells to stimulate IL-1β secretion. The supernatants were recovered, and the amount of IL-1β was measured by ELISA (eBiosciences, 88-7013). NLRP3, pro-Caspase-1, Caspase-1 and pro-IL-1β protein levels were assessed on cell lysates by western blot analysis.

### Fluorescence resonance energy transfer (FRET)

Peritoneal macrophages (10^6^) or murine RAW 264.7 MPs (4 × 10^5^) were seeded overnight in DMEM containing 10% FBS, on round 15 mm cover glass. Cells were weaned off FBS for 2 h with DMEM containing 0.2% BSA. Cells were treated with MPE-001 (10^−7^ M) and stimulated with the TLR2 agonist R-FSL1 (300 ng/mL) for 5 min. The TLR2 (Biorbyt, orb191498) and CD36 (Abcam, ab800080) antibodies were respectively coupled to energy donor Cy3 (ab188287) and energy acceptor Cy5 (ab188288) dyes according to manufacturer’s instructions, then added to fixed cells in a 1:1 mixture of donor/acceptor labeled antibodies. After the incubation period, cells were washed 3 times with PBS and mounted on microscope slides using antifade reagent (Molecular Probes # P36941). FRET efficiency was assessed using a confocal microscope LSM-700 (Zeiss, Oberkochen, Germany) with the acceptor photobleaching method.

### Retinal tissue preparation and immunofluorescence staining

Human and mouse eyes were fixed in 4% PFA and cryoprotected using 30% sucrose. They were embedded in optimal cutting temperature (OCT) compound (Leica, Wetzlar, Germany), frozen in liquid nitrogen, and stored at −80 °C. Frozen sections (10 μm thick) were cut in a cryostat (Leica CM 3050 S) and mounted on gelatin-coated slides for immunofluorescence analysis. For flat mounts, human and mouse eyes were fixed in 4% PFA for 15 min at room temperature and sectioned at the limbus; the anterior segments were discarded. The posterior eye cups consisting of neuroretina/RPE/choroid/sclera complex were collected and the neuroretina was carefully detached from RPE/choroid/sclera to be prepared separately for experiments.

For immunofluorescence, central retina sections, neuroretina and RPE/choroid flat mounts were treated with PBS solution containing 0.1% Triton x100 and 10% FBS or 5% BSA (blocking buffer) for 45 min. Specimens were incubated overnight at 4 °C with primary antibodies diluted in blocking buffer. Immunofluorescence was performed using polyclonal rabbit anti-CD36 (Novus Biologicals, NB400-145), monoclonal mouse anti-CD36 (Abcam, ab125116), monoclonal mouse anti-TLR2 (Invivogen, mab-mtlr2), polyclonal rabbit anti-TLR2 (Biobyt, orb191498), polyclonal rabbit anti-IBA-1, monoclonal rat anti-F4/80 (Abcam, ab6640), polyclonal rabbit anti-iNOS, rat anti-mouse IL-12 PE, polyclonal rabbit anti-GFAP, polyclonal rabbit anti-Opsin-blue antibodies. RPE cells were counter stained with rhodamine phalloidin (Biotium, 00027) and cone photoreceptors were stained with fluorescein peanut agglutinin. The corresponding secondary antibodies: AlexaFluor 488-, 594-conjugated antibodies, and 647-conjugated antibodies were used to reveal the primary antibodies, and sections were counterstained with DAPI. The retinal section analysis was performed exclusively in the central retina and peripheral retina was excluded. Central retina sections and flat mounts were analyzed using confocal microscope (Olympus FluoView 1000, Richmond Hill, ON, Canada). All immunostainings were repeated at least three times and staining that omitted the primary antibody served as negative control.

### MP and retinal explant incubation

BMDM were respectively left untreated or co-stimulated with 300 ng/mL R-FSL1 and 20 ng/mL IFNγ for 24 h with or without MPE-001 (10^−7^ M). To study the role of PPAR-γ in the cytoprotective effect of MPE-001 in apoptosis of photoreceptors induced by activated MPs, PPAR-γ inhibitor (10^−6^ M) (GW9662) was added to BMDM treated with MPE-001 for 24 h. Culture media was removed and the BMDM were cultured with fresh DMEM/10% FBS for 24 h, and the supernatant was used as the conditioned media (CM). Neuroretina explants were generated from 12-16-week-old C57BL/6J mice eyes. The neuroretina was carefully detached from RPE/choroid/sclera to be incubated for 18 h with the CM or on DMEM with BMDM prepared as mentioned in the previous section. To reveal the role of IL-1β released in CM-induced photoreceptor apoptosis, the CM was incubated or not with 150 ng/mL of anti-IL-1β neutralizing antibody (Abcam, 9722) for 15 min at room temperature. After 18 h of stimulation, the explants were carefully removed, and the detection of apoptotic cells was performed using the terminal deoxynucleotidyl transferase dUTP nick end labeling (TUNEL) assay.

### Quantification of activated MPs in the subretinal space

IBA-1–stained RPE cells were counted on flat mounts from control and illuminated mice injected daily with 0.9% NaCl or MPE-001 (289 nmol/kg). Cell numbers were expressed as the mean number of IBA-1–positive cells per mm^2^.

### Quantification of photoreceptor layer thickness

At least three centrals retinal cryosections (with optic nerve) per eye were used to measure the thickness of photoreceptor layers. Twelve measurements per central retinal section were performed at defined distances of the optic nerve. Analysis of layer thickness was performed using ImageJ software (http://imagej.nih.gov/). The area under the curve was integrated using the statistical analysis program (Prism software version 5.01; GraphPad software).

### Laser-capture microdissection

Eyes were enucleated and immediately embedded in OCT compound and snap frozen in liquid nitrogen. Sagittal sections (20 μm) were analyzed on MembraneSlide 1.0 PEN nuclease free slides (Zeiss). Central retina sections were laser microdissected with the Zeiss Palm Microbeam laser microscope system Observer Z1 (Zeiss, Oberkochen, Germany). The retinal section analysis was performed exclusively in the central retina and peripheral retina was excluded. Isolated retinal mRNA was transcribed into cDNA for quantitative real-time PCR analysis.

### TUNEL assay

Neuro-retinal flat mounts were fixed in 4% PFA for 30 min, washed with PBS, post-fixed in frozen acetic acid for 30 min, and washed with PBS. Neuro-retinal flat mounts and retinal cryosections were permeabilized with 0.1% sodium citrate and 0.1% triton for 2 min on ice, and washed with PBS. Samples were incubated with the terminal deoxynucleotidyl transferase (TdT) and fluorescein-dUTP using the *In-Situ* Cell Death Detection Kit (Roche) for 60 min at 37 °C in a humid chamber in the dark. The reaction was stopped by washing the slides three times with PBS. Nuclei were counterstained with DAPI. Samples were analyzed using confocal microscopy (Olympus FluoView 1000, Richmond Hill, ON, Canada).

### Electroretinography

Electroretinographs (ERGs) were recorded from WT and age-matched CD36^−/−^ mice on an Espion ERG Diagnosys apparatus equipped with a ColorDome Ganzfeld stimulator (Diagnosys LLC, Lowell, MA). Mice were dark adapted overnight and anesthetized intraperitoneally with an aqueous solution containing a mixture of ketamine (100 mg/kg) and xylazine (20 m/kg). Pupils were dilated using atropine and phenylephrine. A drop of methylcellulose was placed on the corneal surface to prevent corneal dehydration. Mouse body temperature was maintained at 37 °C using a heated water pad.

Flash scotopic ERGs were measured using corneal DTL Plus electrodes (Diagnosys LLC). The electrodes were placed on the surface of the cornea. A needle electrode on the forehead served as the reference electrode. Another needle grounding electrode was inserted into the tail skin. Scotopic responses were simultaneously stimulated from both eyes of the dark-adapted animals at the following increasing light intensities: 0.5, 1.0, 3.0 and 10.0 candela*second/meter² (cd*s/m²). Ten waveform responses were averaged with an inter-stimulus interval (ISI) of 5 seconds (for 0.5 cd*s/m²) or 20 seconds (for 1, 3 and 10 cd*s/m²). All procedures were performed in a dark room under dim red-light illumination. The amplitude and latency of the major ERG components were measured with the Espion software (Diagnosys LLC). ERG results were recorded at the optimal light intensity of 3.0 cd*s/m². The ERG a-wave amplitudes were measured from the baseline to the primary negative peak and the b-wave amplitudes were measured from the trough of the a-wave to the maximum of the fourth positive peak.

### Bioenergetics

Polarized M0 or M1-BMDM treated or not with MPE-001, or peritoneal macrophages isolated from MPE-001- or NaCl-treated mice were seeded at 2.5 × 10^5^ in Seahorse plates. MPE-001 was injected directly to the Seahorse plate on M1-BMDM. Real-time analysis of oxygen consumption rate (OCR) and extracellular acidification rate (ECAR) were analyzed with an XF-24 Extracellular Flux Analyzer (Seahorse Bioscience, North Billerica, Billerica, MA, USA). Uncoupled respiration (proton leak) represents fraction of total respiration that is insensitive to oligomycin treatment (1μM). Coupled respiration is calculated by substracting proton leak reaction from total respiration and represents reaction coupled to ATP synthesis. The contribution of non-mitochondrial respiration is also substracted in these experiments.

### Metabolite quantification by GC-MS

Following treatments, MPs plated in 6-well plates were rinsed 3 times with 9 g/L NaCl solution (4 °C), quenched with 1.2 mL dry ice-cooled 80% MeOH, and stored at −80 °C. Samples were treated by sonication using the bioruptor (Diagenode, Denville, NJ, USA) for 10 min at the highest setting, with pulses and rests of 30 sec. Samples were cleared by centrifugation, and 800 ng D_27_-myristic acid in pyridine was added as an internal standard. Supernatants were dried up overnight in a vacuum pump concentrator (Labconco, Kansas City, MO, USA) set at −4 °C. Pellets were resuspended in 10 mg/mL methoxyamine hydrochloride/pyridine and subjected to methoximation for 30 min at 70 °C and silylation with N-tert-Butyldimethylsilyl-N-methyltrifluoroacetamide (MTBSTFA) + 1% t-BDMCS for 1 h at 70 °C. Internal standards and derivatization reagents were from Millipore Sigma (Oakville, ON, CA). Samples (1 µL) were injected in splitless mode in a 5975 C GC-MS configured with a DB-5MS + DG (30 m × 259 µm × 0.25 µm) capillary column (Agilent, Santa Clara, CA, USA). Inlet temperature was set to 280 °C and the carrier gas was helium. The flow rate was set to lock the internal standard elution at 17.94 min. The quadrupole was set at 150 °C and the GC-MS interface at 285 °C. The oven program started at 60 °C for 1 min, then temperature was raised by 10 °C/min until 320 °C. Bake-out was at 320 °C (10 min). Data was acquired in scan mode and showed no saturation. All metabolites measured were validated using authentic standards (Sigma Millipore). Data analysis was done using the ChemStation software (Agilent). Relative metabolite concentrations were obtained by correcting the peak areas of quantifying ions with those of D_27_-myristic acid, and by dividing this ratio with the average protein content associated with paired BMDM plates, thus providing data with arbitrary units. Principal component analyses (PCA) were performed with MetaboAnalyst 4.0^79,80^ using data obtained from 2 independent experiments, each conducted with 2-3 independent BMDM cultures. Data was uploaded as.csv files in MetaboAnalyst prior to the generation of PCA plots (containing 2 principal components).

### Quantitative real-time PCR

RNA was run on a 2100 Bioanalyzer using a Nano RNA chip to verify its integrity. Total RNA was treated with DNase and reverse transcribed using the Maxima First Strand cDNA synthesis kit with ds DNase (Thermo Scientific). Gene expression was determined using assays designed with the Universal Probe Library from Roche (www.universalprobelibrary.com), and when no probe was available, a SYBR Green assay was designed. For all qPCR assay, a standard curve was performed to ensure that the efficacy of the assay is between 90% and 110%. For UPL assays, qPCR reactions were performed using Taqman Advanced master mix (Life Technologies), 2 µM of each primer and 1 µM of the corresponding UPL probe. For SYBR green assays, a melt curve was performed to ensure only a single product was amplified, and qPCR reactions were performed using Fast SYBR Green Master Mix (Wisent) and 2 µM of each primer. The Viia7 qPCR instrument (Life Technologies) was used to detect the amplification level and was programmed with an initial step of 20 sec at 95 °C, followed by 40 cycles of 1 sec at 95 °C and 20 sec at 60 °C. Relative expression (RQ = 2^−ΔΔCT^) was calculated using the Expression Suite software (Life Technologies), and normalization was done using both glyceraldehyde 3-phosphate dehydrogenase (*Gapdh*) and actin beta (*Actb*).

### Flow cytometry

Polarized M1 or M0 BMDM (10^6^/well) were incubated with MPE-001 (10^−7^ M) for 24 h. Cells were then washed twice with cold PBS and incubated for 10 min at 4 °C with anti-mouse CD16/32. Cells were then incubated for 30 min at 4 °C with anti-mouse APC/Cy7-F4/80 (1:100), BV421-MHCII (1:400) and PE-Cy5-CD86 (1:800) all diluted in FACS buffer. For PE-CD206, intracellular staining was performed using Cytofix/cytoperm buffer for 20 min at 4 °C. Cells were washed three times with FACS buffer then fixed in 4% PFA for 15 min on ice and analyzed by flow cytometry using a Canto II flow cytometer (Becton, Dickinson, Franklin Lakes, NJ).

### Phagocytosis assay

Phagocytosis was assessed by a modified protocol from that previously described^81^. Briefly, microspheres (carboxylate-modified FluoSpheresTM, coupled to yellow-green fluorescence, 2 μm, F8827, Invitrogen) were pre-coated with a 1% solution of BSA at pH 9.0 for 30 min at 37 °C. Coated albumin microspheres were washed three times with PBS before use in the assay. Polarized M1-BMDM (10^6^/well, in 6-well plates) were incubated with the coated microspheres at a ratio of 50 beads per 1 cell (50/1 ratio) for 90 min at 37 °C or at 4 °C in a final volume of 2 mL complete media. MPE-001 (10^−6^ M) was added to the cells 30 min before the addition of microspheres. Cells were then washed twice with cold PBS, fixed in 4% PFA for 15 min on ice, and analyzed by flow cytometry using a Canto II flow cytometer (Becton, Dickinson, Franklin Lakes, NJ, USA).

### Statistical analysis

Results are expressed as mean ± SEM. Statistical significance was calculated with unpaired Student’s *t*-test to compare 2 conditions. Comparisons between groups were performed on normally distributed data using one-way ANOVA and Student-Newman-Keuls (SNK) post-hoc test. Statistical significance was set based on *P value*: *P < 0.05, **P < 0.01, ***P < 0.001. All experiments were repeated at least 3 times. Statistical analysis was performed using GraphPad Prism 7.0 software.

### Study approval

All experimental procedures were approved by the Institutional Animal Ethics Committee in strict accordance with the Canadian Council on Animal Care guidelines and the guide for the Use of Laboratory Animals published by the US National Institutes of Health. The human clinical protocol and informed consent forms were approved by the CHU Sainte-Justine ethics committee and adhered to the tenets of the Declaration of Helsinki. A signed informed consent was obtained from all subjects before enrollment in the study.

## Supplementary information

Dataset 1

## References

[1] Xu H, Chen M, Forrester JV (2009). Para-inflammation in the aging retina. Prog. Retin. Eye Res..

[2] Chow A, Brown BD, Merad M (2011). Studying the mononuclear phagocyte system in the molecular age. Nat. Rev. Immunol..

[3] Guillonneau X (2017). On phagocytes and macular degeneration. Prog. Retin. Eye Res..

[4] Dong, Y. et al. Insights from Genetic Model Systems of Retinal Degeneration: Role of Epsins in Retinal Angiogenesis and VEGFR2 Signaling. *J Nat Sci* **3** (2017). PMC5303005 28191500

[5] Whitcup SM, Nussenblatt RB, Lightman SL, Hollander DA (2013). Inflammation in retinal disease. Int J Inflam.

[6] Di Gioia M, Zanoni I (2015). Toll-like receptor co-receptors as master regulators of the immune response. Mol. Immunol..

[7] van Bergenhenegouwen J (2013). TLR2 & Co: a critical analysis of the complex interactions between TLR2 and coreceptors. J. Leukoc. Biol..

[8] Feng L (2017). A Proinflammatory Function of Toll-Like Receptor 2 in the Retinal Pigment Epithelium as a Novel Target for Reducing Choroidal Neovascularization in Age-Related Macular Degeneration. Am. J. Pathol..

[9] Reis A (2014). Neuroretinal dysfunction with intact blood-retinal barrier and absent vasculopathy in type 1 diabetes. Diabetes.

[10] Huh HY, Pearce SF, Yesner LM, Schindler JL, Silverstein RL (1996). Regulated expression of CD36 during monocyte-to-macrophage differentiation: potential role of CD36 in foam cell formation. Blood.

[11] Coraci IS (2002). CD36, a class B scavenger receptor, is expressed on microglia in Alzheimer’s disease brains and can mediate production of reactive oxygen species in response to beta-amyloid fibrils. Am. J. Pathol..

[12] Beutler B (2006). Genetic analysis of host resistance: Toll-like receptor signaling and immunity at large. Annu. Rev. Immunol..

[13] Abe T (2010). Key role of CD36 in Toll-like receptor 2 signaling in cerebral ischemia. Stroke.

[14] Kawai T, Akira S (2010). The role of pattern-recognition receptors in innate immunity: update on Toll-like receptors. Nat. Immunol..

[15] Smolinska MJ, Page TH, Urbaniak AM, Mutch BE, Horwood NJ (2011). Hck tyrosine kinase regulates TLR4-induced TNF and IL-6 production via AP-1. J. Immunol..

[16] Zhang J, Mulumba M, Ong H, Lubell WD (2017). Diversity-Oriented Synthesis of Cyclic Azapeptides by A(3) -Macrocyclization Provides High-Affinity CD36-Modulating Peptidomimetics. Angew. Chem. Int. Ed. Engl..

[17] Saha S, Shalova IN, Biswas SK (2017). Metabolic regulation of macrophage phenotype and function. Immunol. Rev..

[18] Gupta N, Brown KE, Milam AH (2003). Activated microglia in human retinitis pigmentosa, late-onset retinal degeneration, and age-related macular degeneration. Exp. Eye Res..

[19] Chinnery HR (2012). Accumulation of murine subretinal macrophages: effects of age, pigmentation and CX3CR1. Neurobiol. Aging.

[20] Sennlaub F (2013). CCR2(+) monocytes infiltrate atrophic lesions in age-related macular disease and mediate photoreceptor degeneration in experimental subretinal inflammation in Cx3cr1 deficient mice. EMBO Mol. Med..

[21] Eandi, C. M. *et al*. Subretinal mononuclear phagocytes induce cone segment loss via IL-1beta. *Elife* **5**, 10.7554/eLife.16490 (2016). 10.7554/eLife.16490 PMC4969036 27438413

[22] de Raad S, Szczesny PJ, Munz K, Reme CE (1996). Light damage in the rat retina: glial fibrillary acidic protein accumulates in Muller cells in correlation with photoreceptor damage. Ophthalmic Res..

[23] Bringmann A, Reichenbach A (2001). Role of Muller cells in retinal degenerations. Front. Biosci..

[24] Levy O (2015). Apolipoprotein E promotes subretinal mononuclear phagocyte survival and chronic inflammation in age-related macular degeneration. EMBO Mol. Med..

[25] Combadiere C (2007). CX3CR1-dependent subretinal microglia cell accumulation is associated with cardinal features of age-related macular degeneration. J. Clin. Invest..

[26] Cruz-Guilloty F (2013). Infiltration of proinflammatory m1 macrophages into the outer retina precedes damage in a mouse model of age-related macular degeneration. Int J Inflam.

[27] Scholz R (2015). Minocycline counter-regulates pro-inflammatory microglia responses in the retina and protects from degeneration. J. Neuroinflammation.

[28] Motoi Y (2014). Lipopeptides are signaled by Toll-like receptor 1, 2 and 6 in endolysosomes. Int. Immunol..

[29] Hoebe K (2005). CD36 is a sensor of diacylglycerides. Nature.

[30] Jimenez-Dalmaroni MJ (2009). Soluble CD36 ectodomain binds negatively charged diacylglycerol ligands and acts as a co-receptor for TLR2. PLoS One.

[31] Triantafilou M (2006). Membrane sorting of toll-like receptor (TLR)-2/6 and TLR2/1 heterodimers at the cell surface determines heterotypic associations with CD36 and intracellular targeting. J. Biol. Chem..

[32] Okusawa T (2004). Relationship between structures and biological activities of mycoplasmal diacylated lipopeptides and their recognition by toll-like receptors 2 and 6. Infect. Immun..

[33] Schwandner R, Dziarski R, Wesche H, Rothe M, Kirschning CJ (1999). Peptidoglycan- and lipoteichoic acid-induced cell activation is mediated by toll-like receptor 2. J. Biol. Chem..

[34] Darveau RP (2004). Porphyromonas gingivalis lipopolysaccharide contains multiple lipid A species that functionally interact with both toll-like receptors 2 and 4. Infect. Immun..

[35] Ozinsky A (2000). The repertoire for pattern recognition of pathogens by the innate immune system is defined by cooperation between toll-like receptors. Proc. Natl. Acad. Sci. USA.

[36] Re F, Strominger JL (2003). Separate functional domains of human MD-2 mediate Toll-like receptor 4-binding and lipopolysaccharide responsiveness. J. Immunol..

[37] Ng TF, Streilein JW (2001). Light-induced migration of retinal microglia into the subretinal space. Invest. Ophthalmol. Vis. Sci..

[38] Zeiss CJ, Johnson EA (2004). Proliferation of microglia, but not photoreceptors, in the outer nuclear layer of the rd-1 mouse. Invest. Ophthalmol. Vis. Sci..

[39] Zhang C (2005). Activation of microglia and chemokines in light-induced retinal degeneration. Mol. Vis..

[40] Sheedy FJ (2013). CD36 coordinates NLRP3 inflammasome activation by facilitating intracellular nucleation of soluble ligands into particulate ligands in sterile inflammation. Nat. Immunol..

[41] Hu SJ (2015). Upregulation of P2RX7 in Cx3cr1-Deficient Mononuclear Phagocytes Leads to Increased Interleukin-1beta Secretion and Photoreceptor Neurodegeneration. J. Neurosci..

[42] Mills CD, Kincaid K, Alt JM, Heilman MJ, Hill AM (2000). M-1/M-2 macrophages and the Th1/Th2 paradigm. J. Immunol..

[43] Park YM, Febbraio M, Silverstein RL (2009). CD36 modulates migration of mouse and human macrophages in response to oxidized LDL and may contribute to macrophage trapping in the arterial intima. J. Clin. Invest..

[44] Kannan Y, Sundaram K, Aluganti Narasimhulu C, Parthasarathy S, Wewers MD (2012). Oxidatively modified low density lipoprotein (LDL) inhibits TLR2 and TLR4 cytokine responses in human monocytes but not in macrophages. J. Biol. Chem..

[45] Datta G (2015). Bioenergetic programming of macrophages by the apolipoprotein A-I mimetic peptide 4F. Biochem. J..

[46] Bassaganya-Riera J, Misyak S, Guri AJ, Hontecillas R (2009). PPAR gamma is highly expressed in F4/80(hi) adipose tissue macrophages and dampens adipose-tissue inflammation. Cell. Immunol..

[47] Huss JM, Kelly DP (2004). Nuclear receptor signaling and cardiac energetics. Circ. Res..

[48] Joly S (2009). Cooperative phagocytes: resident microglia and bone marrow immigrants remove dead photoreceptors in retinal lesions. Am. J. Pathol..

[49] Anderson DH (2010). The pivotal role of the complement system in aging and age-related macular degeneration: hypothesis re-visited. Prog. Retin. Eye Res..

[50] Hennessy EJ, Parker AE, O’Neill LA (2010). Targeting Toll-like receptors: emerging therapeutics?. Nat Rev Drug Discov.

[51] Gao W, Xiong Y, Li Q, Yang H (2017). Inhibition of Toll-Like Receptor Signaling as a Promising Therapy for Inflammatory Diseases: A Journey from Molecular to Nano Therapeutics. Front. Physiol..

[52] Demers A (2004). Identification of the growth hormone-releasing peptide binding site in CD36: a photoaffinity cross-linking study. Biochem. J..

[53] Yang X (2017). CD36 in chronic kidney disease: novel insights and therapeutic opportunities. Nat Rev Nephrol.

[54] Chen YP (2016). Palmitic acid interferes with energy metabolism balance by adversely switching the SIRT1-CD36-fatty acid pathway to the PKC zeta-GLUT4-glucose pathway in cardiomyoblasts. J. Nutr. Biochem..

[55] Conroy H, Marshall NA, Mills KH (2008). TLR ligand suppression or enhancement of Treg cells? A double-edged sword in immunity to tumours. Oncogene.

[56] Suzuki M (2012). Chronic photo-oxidative stress and subsequent MCP-1 activation as causative factors for age-related macular degeneration. J. Cell Sci..

[57] Areschoug T, Gordon S (2009). Scavenger receptors: role in innate immunity and microbial pathogenesis. Cell. Microbiol..

[58] Cao D (2016). CD36 regulates lipopolysaccharide-induced signaling pathways and mediates the internalization of Escherichia coli in cooperation with TLR4 in goat mammary gland epithelial cells. Sci. Rep..

[59] Barton GM, Medzhitov R (2003). Toll-like receptor signaling pathways. Science.

[60] Ko MK, Saraswathy S, Parikh JG, Rao NA (2011). The role of TLR4 activation in photoreceptor mitochondrial oxidative stress. Invest. Ophthalmol. Vis. Sci..

[61] Yakubenko VP, Bhattacharjee A, Pluskota E, Cathcart MK (2011). alphaMbeta(2) integrin activation prevents alternative activation of human and murine macrophages and impedes foam cell formation. Circ. Res..

[62] Huang W, Febbraio M, Silverstein RL (2011). CD9 tetraspanin interacts with CD36 on the surface of macrophages: a possible regulatory influence on uptake of oxidized low density lipoprotein. PLoS One.

[63] Wintergerst ES, Jelk J, Rahner C, Asmis R (2000). Apoptosis induced by oxidized low density lipoprotein in human monocyte-derived macrophages involves CD36 and activation of caspase-3. Eur. J. Biochem..

[64] Seimon TA (2010). Atherogenic lipids and lipoproteins trigger CD36-TLR2-dependent apoptosis in macrophages undergoing endoplasmic reticulum stress. Cell Metab..

[65] Mantovani A (2004). The chemokine system in diverse forms of macrophage activation and polarization. Trends Immunol..

[66] Martinez FO, Gordon S (2014). The M1 and M2 paradigm of macrophage activation: time for reassessment. F1000Prime Rep.

[67] Vats D (2006). Oxidative metabolism and PGC-1beta attenuate macrophage-mediated inflammation. Cell Metab..

[68] Tannahill GM (2013). Succinate is an inflammatory signal that induces IL-1beta through HIF-1alpha. Nature.

[69] Bujold K (2009). CD36-mediated cholesterol efflux is associated with PPARgamma activation via a MAPK-dependent COX-2 pathway in macrophages. Cardiovasc. Res..

[70] Shu Y (2016). Phosphorylation of PPARgamma at Ser84 promotes glycolysis and cell proliferation in hepatocellular carcinoma by targeting PFKFB4. Oncotarget.

[71] Guo B, Huang X, Lee MR, Lee SA, Broxmeyer HE (2018). Antagonism of PPAR-gamma signaling expands human hematopoietic stem and progenitor cells by enhancing glycolysis. Nat. Med..

[72] Ye F (2011). Peroxisome proliferator-activated receptor gamma (PPARgamma) mediates a Ski oncogene-induced shift from glycolysis to oxidative energy metabolism. J. Biol. Chem..

[73] Reddy RC (2008). Immunomodulatory role of PPAR-gamma in alveolar macrophages. J. Investig. Med..

[74] Karlstetter M (2015). Retinal microglia: just bystander or target for therapy?. Prog. Retin. Eye Res..

[75] Gao J (2015). NLRP3 inflammasome: activation and regulation in age-related macular degeneration. Mediators Inflamm..

[76] Gurung P (2015). Chronic TLR Stimulation Controls NLRP3 Inflammasome Activation through IL-10 Mediated Regulation of NLRP3 Expression and Caspase-8 Activation. Sci. Rep..

[77] Combadiere C (2003). Decreased atherosclerotic lesion formation in CX3CR1/apolipoprotein E double knockout mice. Circulation.

[78] Febbraio M (1999). A null mutation in murine CD36 reveals an important role in fatty acid and lipoprotein metabolism. J. Biol. Chem..

[79] Chong J (2018). MetaboAnalyst 4.0: towards more transparent and integrative metabolomics analysis. Nucleic Acids Res.

[80] Xia J, Psychogios N, Young N, Wishart DS (2009). MetaboAnalyst: a web server for metabolomic data analysis and interpretation. Nucleic Acids Res.

[81] Babaev VR (2011). Selective macrophage ascorbate deficiency suppresses early atherosclerosis. Free Radic. Biol. Med..

