# Supplementary material for: Immunometabolic modulation of retinal inflammation by CD36 ligand
Source: Sci Rep. 2019 Sep 9;9:12903. doi: 10.1038/s41598-019-49472-8 (PMC6733801; doi:10.1038/s41598-019-49472-8)
Supplement: Supplementary file 1 — Dataset 1 [file 41598_2019_49472_MOESM1_ESM.pdf]

Supplemental Materials for

Immunometabolic modulation of retinal inflammation by CD36 ligand

Authors: Katia Mellal†, Samy Omri†, Mukandila Mulumba, Houda Tahiri, Carl Fortin, Marie-France Dorion, Hung Pham, Yesica Garcia Ramos, Jinqiang Zhang, Sheetal Pundir, Jean-Sébastien Joyal, Jean-François Bouchard, Florian Sennlaub, Maria Febbraio, Pierre Hardy, Simon-Pierre Gravel, Sylvie Marleau, William D Lubell, Sylvain Chemtob* and Huy Ong*

* Corresponding author. Email: sylvain.chemtob@umontreal.ca (S.C.), huy.ong@umontreal.ca (H.O.).

† These authors contributed equally to this work.

The Supplemental Material file includes:

Fig. S1. MPE-001 downregulates subretinal inflammation and photoreceptor degeneration in light-exposed CX3CR1$^{-/-}$ and CX3CR1$^{-/-}$/CD36$^{-/-}$ mice.

Fig. S2. Effect of MPE-001 on R-FSL1-stimulated release of cytokines in human macrophages.

Fig. S3. Colocalization of CD36 and TLR2 in lipid rafts and MPE-001 effect on TLR2-signaling

Fig. S4. MPE-001 exerts no effect on macrophage phagocytosis.

Fig. S5. Azapeptide MPE-001 decreases caspase-1 expression in outer retina.

Fig. S6. Immuno-metabolic effects of MPE-001 on macrophages.

Fig. S7. Subretinal MPs in CD36+/+/TLR2+/+ in mice and aging human retina.

Table S1. Percentage of pro-inflammatory cytokine/chemokine reduction in response to MPE-001-treatment in TLR2-stimulated peritoneal macrophages.

Table S2. Percentage of IL-1β reduction in response to MPE-001-treatment on R-FSL1-stimulated peritoneal macrophages.

Table S3**.** Primers ID and sequences used for quantitative RT-PCR

Supplemental figures

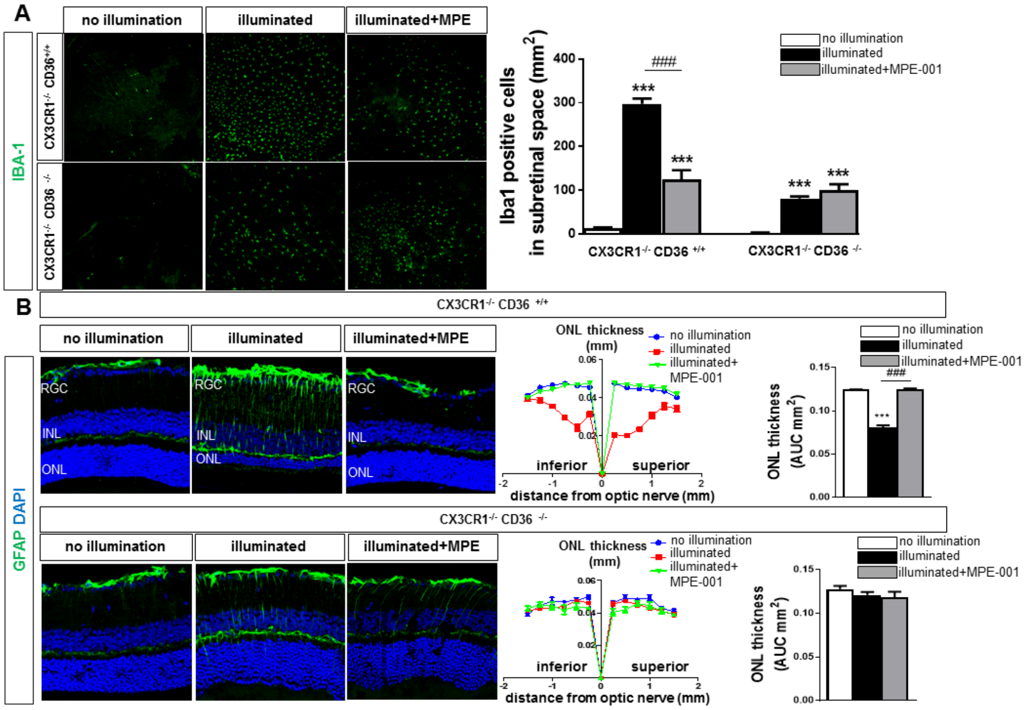

Figure S1. MPE-001 downregulates subretinal inflammation and photoreceptor degeneration in light-exposed CX3CR1$^{-/-}$ and CX3CR1$^{-/-}$/CD36$^{-/-}$ mice.
(A) Evidence of CD36 involvement in light-induced accumulation of subretinal macrophages. Representative RPE/Chr/Scl flat mounts, illustrating the accumulation of IBA-1+ cells (green) in the subretinal space of CX3CR1$^{-/-}$ or CX3CR1$^{-/-}$ /CD36$^{-/-}$ mice illuminated with bluelight for 5 days and treated or not with 289 nmol/kg per day of MPE-001 from day 1 to day 7. Quantification of IBA-1+ cells in the subretinal space of mice unexposed, illuminated with blue light, and treated or not with MPE-001 (n = 3-4 mice per group). **(B)** Evidence of CD36 involvement in light-induced degeneration of ONL. GFAP staining (green) in retinal cryosections from CX3CR1$^{-/-}$ or CX3CR1$^{-/-}$ /CD36$^{-/-}$ mice exposed or not to blue light and treated or not with MPE-001. ONL thickness measurements and spider graph representations showing both side of the optic nerve from CX3CR1$^{-/-}$/CD36$^{+/+}$ or CX3CR1$^{-/-}$/CD36$^{-/-}$ mice unexposed or exposed to blue light for 5 days and treated or not with 289 nmol/kg per day of MPE-001 from day 1 to day 7. Area under the curves from ONL thickness measurement. One-way ANOVA with Newman-Keuls for multiple comparisons was performed. *** $P < 0.001$ vs no illumination, ### $P < 0.001$ vs illuminated group. Data are shown as mean ± S.E.M. ONL: Outer Nuclear Layer, INL: Inner Nuclear Layer, GCL: Ganglion Cell Layer.

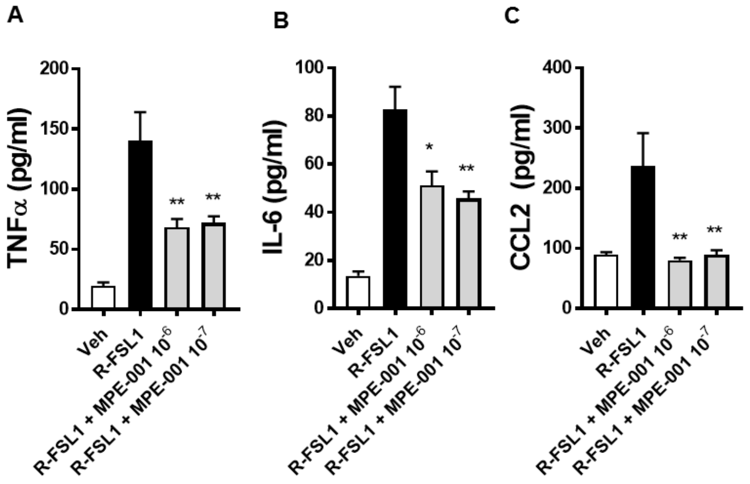

Figure S2. Effect of MPE-001 on R-FSL1-stimulated release of cytokines in human macrophages.

(A - C) TNFα, IL-6 and CCL2 in supernatants of human macrophages after 4 h stimulation with 300 ng/ml R-FSL1 in the presence of MPE-001 or vehicle. One-way ANOVA with Newman-Keuls post-test for multiple comparisons was performed. Data are representative of 2 independent experiments (n = 4 per group). * $P < 0.05$, ** $P < 0.01$ vs R-FSL1. Data are shown as mean ± S.E.M.

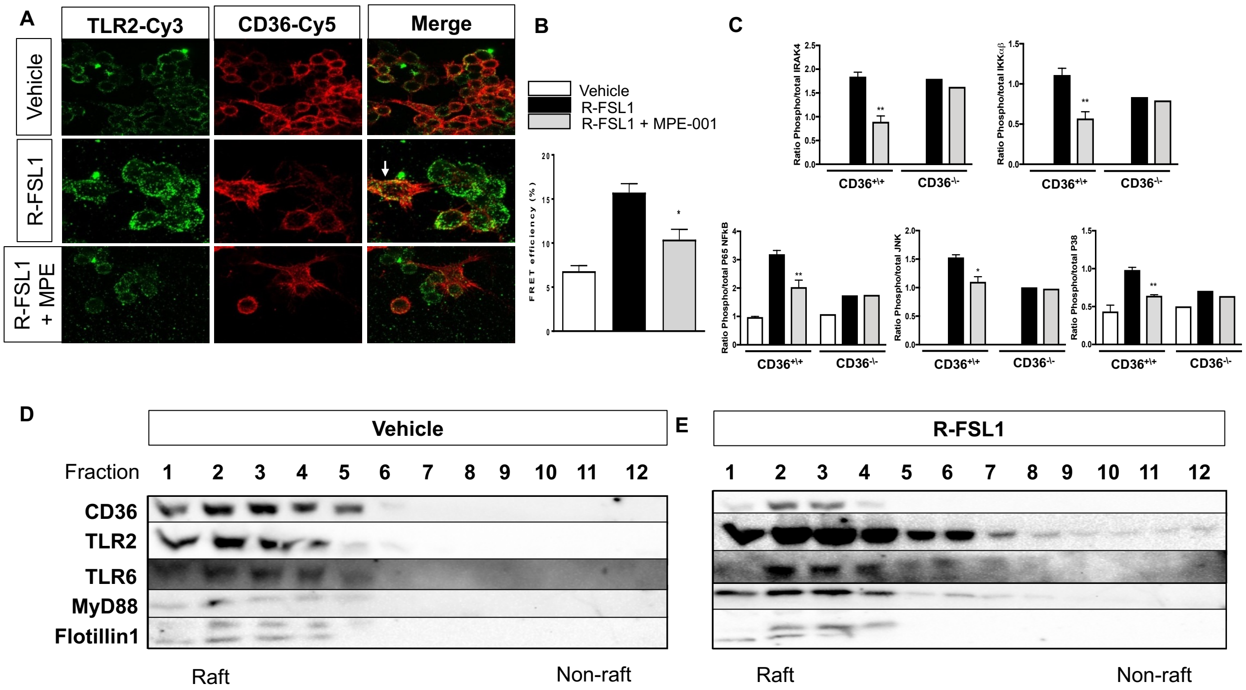

Figure S3. Colocalization of CD36 and TLR2 in lipid rafts and MPE-001 effect on TLR2-signaling.

(A) RAW macrophages were stimulated with 300 ng/ml R-FSL1 and treated with 10^{-7} M MPE-001 for 10 min. Azapeptide MPE-001 disrupted the interaction between CD36 labeled with Cy5 (red), TLR2 labeled with Cy3 (green) and merge shown in yellow as assessed by fluorescence resonance energy transfer (FRET), measured using LSM-700 (Zeiss) confocal microscope. **(B)** Quantification of FRET efficiency expressed as the percentage of energy transfer. Data are representative of 3 independent experiments (n = 10-30 per group). **(C)** Densitometric analysis data of western blots shown in Fig. 4E. **(D, E)** CD36 and TLR2 colocalization in lipid rafts after R-FSL1 stimulation. Peritoneal macrophages were treated with vehicle **(D)** or stimulated with 300 ng/ml R-FSL1 **(E)** for 5 min and subjected to sucrose density gradient ultracentrifugation. Data are representative of 3 experiments. The relative positions of the raft and non-raft (soluble) fractions are indicated. One-way ANOVA with Newman-Keuls post-test for multiple comparisons was performed. *$P < 0.05$. Data are shown as mean ± S.E.M.

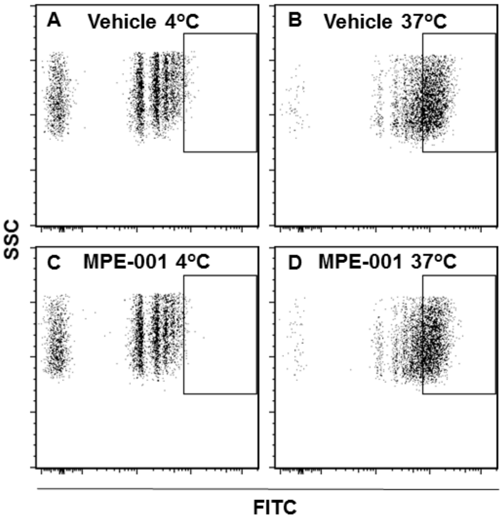

Figure S4. MPE-001 exerts no effect on macrophage phagocytosis.
(A-D) Proinflammatory induced-BMDM were incubated with yellow-green microspheres at 4°C, to determine background values **(A, C)**; or at 37°C, to measure the phagocytosed microspheres **(B, D)**. Phagocytosis is expressed as the relative number of cells that have ingested fluorescent beads in vehicle **(A, B)**, or after MPE-001 treatment **(C, D).** Data are representative of 2 independent experiments.

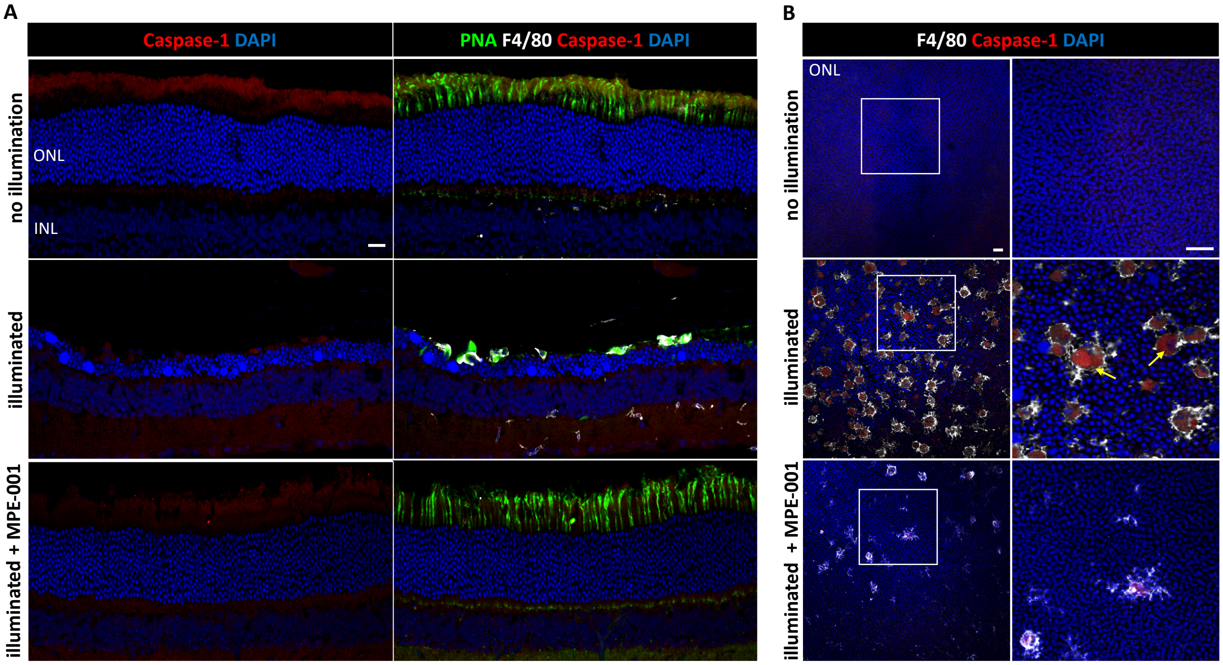

Figure S5. Azapeptide MPE-001 decreases caspase-1 expression in outer retina. Confocal microscopy of retina cryosection **(A)** and neuroretinal flat mounts (photoreceptors side) **(B)** from illuminated $CD36^{+/+}$ mice treated or not with MPE-001 stained with caspase-1 (red), PNA (green) and F4/80 (white); nuclei were counterstained with DAPI (blue). Magnifications of white square show caspase-1 distribution in outer retina. Yellow arrows show caspase-1 expression in subretinal F4/80+ cells. Scale: 10 μm.

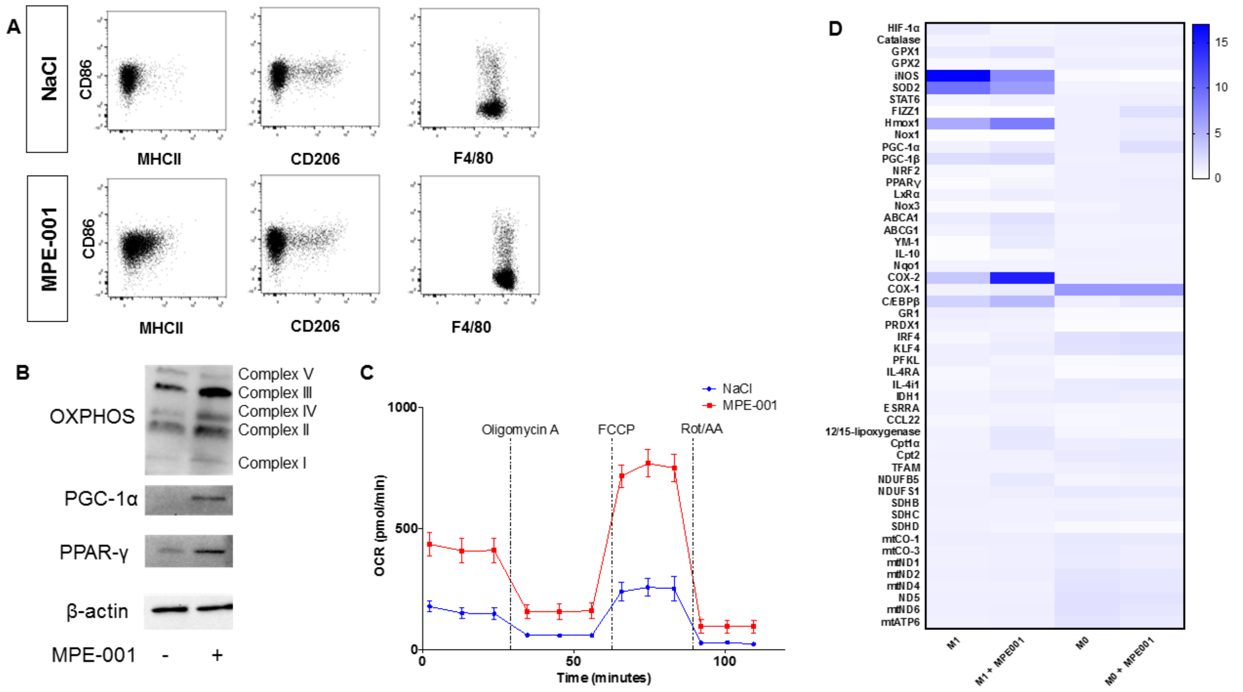

Figure S6. Immuno-metabolic effects of MPE-001 on macrophages.
(A-C) Peritoneal macrophages were isolated from WT mice treated daily *s.c.* injection of MPE-001 (289 nmol/kg) or NaCl for 7 days. **(A)** Phenotypic analysis of isolated peritoneal macrophages by flow cytometry using MHCII, CD86 and CD206 markers. **(B)** Protein expression of mitochondrial electron transport chain (mtETC) complex subunits (OXPHOS), PGC-1α and PPAR-γ. **(C)** OCR analysis of peritoneal macrophages. Data in **A-C** are representative of 4 independent experiments. **(D)** The heat map of 51 genes expression from M1 and M0 BMDM treated with vehicle or MPE-001 (10^{-7} M) for 24 h and analyzed by qPCR (n = 3 per group).

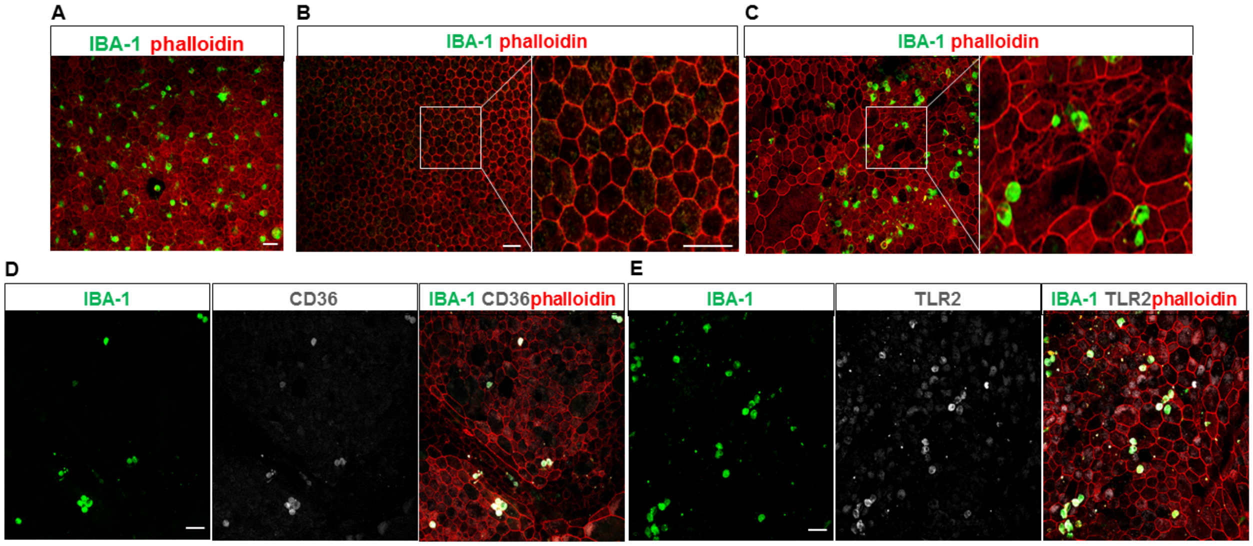

Figure S7 : Subretinal MPs in CD36$^{+/+}$/TLR2$^{+/+}$ in mice and aging human retina. (A-D) Confocal microscopy of RPE flat mounts obtained from illuminated CD36$^{+/+}$/TLR2$^{+/+}$ mouse or aging human donors. **(A)** Double-labeling of subretinal MPs of RPE flat mounts from illuminated mouse showing IBA-1 (green) and phalloidin (for F-actin, red). **(B, C)** Double-labeling of subretinal MPs of RPE flat mounts from human subjects aged 77 **(B)** and 79 **(C)** years old, without and with subretinal inflammation, respectively. Immunofluorescence showing IBA-1 (green) and phalloidin (for F-actin, red). **(C)** White squares magnification (3-fold) show RPE cell morphology (red). **(D)** Triple labeling of subretinal MPs with IBA-1 (green), CD36 (white) and phalloidin (for F-actin, red), of RPE flat mount from a 79-year-old subject with eye inflammation. **(E)** Triple labeling of subretinal MPs with IBA-1 (green), TLR2 (white) and phalloidin (for F-actin, red), of RPE flat mount from a 79-year-old subject with eye inflammation. Scale bar = 100 µm.

Supplementary tables

Table S1. Percentage of pro-inflammatory cytokine/chemokine reduction in response to MPE-001-treatment in TLR2-stimulated peritoneal macrophages.

	TNFα	IL-6	CCL2	IL-12
R-FSL1	38.4 ($P < 0.01$)	37.6 ($P < 0.01$)	41.5 ($P < 0.01$)	39.2 ($P < 0.01$)
LTA	24.5 ($P < 0.01$)	35.0 ($P < 0.01$)	31.9 ($P < 0.01$)	29.1 ($P < 0.01$)
*pg*LPS	38.4 ($P < 0.01$)	42.3 ($P < 0.01$)	30.9 ($P < 0.01$)	33.4 ($P < 0.01$)

Table S2. Percentage of IL-1β reduction in response to MPE-001-treatment on R-FSL1-stimulated peritoneal macrophages.

	IL-1β
MPE-001 10^{-9}	39.2 ($P < 0.05$)
MPE-001 10^{-8}	62.7 ($P < 0.01$)
MPE-001 10^{-7}	74.4 ($P < 0.01$)

Table S3. Primers ID and sequences used for quantitative RT-PCR.

Oligo ID	Gene	UPL Probe	Oligo FWD	Oligo REV	RefSeq	Slope
IR5845	Chil3	88	ggtctgaaagacaagaacactgag	gagaccatggcactgaacg	NM_009892.3	3,2
IR0878	Hif1a	18	catgatggctccctttttca	gtcacctggttgctgcaata	NM_010431.1	3,59
IR1044	Cat	68	gcgaccagatgaagcagtg	gtggtcaggacatcaggtctc	NM_009804.1	3,4
IR1055	Gpx1	2	tttcccgtgcaatcagttc	tcggacgtacttgagggaat	NM_008160.2	3,5
IR1068	Nos2	13	ctttgccacggacgagac	tcattgtactctgagggctgac	NM_010927.1	3,2
IR1084	Sod2	3	gacccattgcaaggaacaa	gtagtaagcgtgctcccacac	NM_013671.3	3,3
IR1262	Stat6	3	tcctggtcacagttcaataagg	caaaccactgccaaaatgtg	NM_009284.1	3,52
IR2537	Retnla	51	ccctccactgtaacgaagactc	cacacccagtagcagtcatcc	NM_020509.3	3,44
IR4021	Ppargc1a	29	gaaagggccaaacagagaga	gtaaatcacacggcgctctt	NM_008904.2, NR_027710.1	3,19
IR4024	Nfe2l2	18	catgatggacttggagttgc	cctccaaaggatgtcaatcaa	NM_010902.3	3,43
IR4352.3	Pparg	67	caagccctttaccacagttga	caggttctactttgatcgcactt	NM_001127330.1, NM_011146.3	3,5
IR5347	Nr1h3	52	gagtgtcgacttcgcaaatg	cggatctgttcttctgacagc	NM_013839.4	3,6
IR6026	Ptgs2	83	caatgtgcaagatccacagc	gtctggagtgggaggcact	NM_011198.4	3,48
IR4022	Ppargc1b	88	gacgtggacgagctttcact	gagcgtcagagcttgctgtt	NM_133249.2	3.40
IR5843	Abca1	26	atggagcagggaagaccac	gtaggccgtgccagaagtt	NM_013454.3	3.2
IR5844	Abcg1	64	tgcttgtcactcaccctatgtaa	ttcccaggataccctgtcc	NM_009593.2	3.3
IR1075	Prdx1	15	gtgagacctgtggctcgac	tgtccatctggcataacagc	NM_011034.2	3.3
IR5846.2	Il10	48	cagccgggaagacaataact	gttgtccagctggtcctttg	NM_010548.2	3,3
IR6027	Cebpd	32	ctgaacgacctatacctcagacc	gcaggtcccaaagaaactagc	NM_007679.4	3,38
IR1062	Gr1	64	gttcctcacgagagccagat	tccagctgaaagaagccatc	NM_010344.3	4
IR1075	Prdx1	15	gtgagacctgtggctcgac	tgtccatctggcataacagc	NM_011034.2	3,3
IR2319	Irf4	26	accccatgacagcaccttat	gggtggcatcatgtagttatga	NM_013674.1	3,59
IR2899	Klf4	62	cgggaagggagaagacact	gagttcctcacgccaacg	NM_010637.3	3,33
IR3771	Cox1	101	cagaccgcaacctaaacaca	ggtgcccaaagaatcagaata	5912286	3,1
IR3773	Cox3	88	cataaatcaagccctactaattaccat	ctgaaatggagaatgatgtttca	NP_904334.1	3,12
IR3774	Nd1	29	acacttattacaacccaagaacacat	tcatattatggctatgggtcagg	NP_904328.1	3,18
IR3775	Nd2	67	ccatcaactcaatctcacttctatg	gaatcctgttagtggtggaagg	NP_904329.1	3,27
IR3776	Nd4	86	gcctaaacgcagggatttatt	gggttcctacatggttttgg	NP_904337.1	3,12
IR3777.2	Nd5	31	agcattcggaagcatctttg	ttgtgaggactggaatgctg	NP_904338.1	3,29
IR3778	Nd6	12	cacaactatatattgccgctaccc	tggtttgggagattggttg	NP_904339.1	3,15
IR3779	Atp6	78	tccataaatctaagtatagccattcca	tttgtgtcggaagcctgtaa		3,25
IR4074	Tfam	94	caaaggatgattcggctcag	aagctgaatatatgcctgcttttc	NM_009360.4	3,3
IR4346	Ndufb5	68	cctggctatcctccagattg	cgcatcagccttcgaact	NM_025316.2	3,4
IR4349	Cox5b	26	gtggtgtccccactgatga	tgaagctgcctttggaggta	NM_009942.2	3,6
IR5344	Cpt2	66	ccaaagaagcagcgatgg	tagagctcaggcagggtga	NM_009949.2	3,4
IR5345	Cd36	75	ccaagctattgcgacatgatt	tctcaatgtccgagacttttca	NM_001159558.1	3,33
IR5353	Cox7b	21	aacgcactaagccgtctcc	catggaaactaggtgccctct	NM_025379.2	3,12
IR5354	Ndufab1	78	tgcagataagaaggatgtgtatgaa	ttcactcttggttgtcagtgtgta	NM_028177.3	3,3
IR5355	Ndufb6	31	agctcgaagcccaggatatt	cttcaatgatgttgatcaggaaa	NM_001033305.2	3,37
IR5356	Ndufs1	60	tgactctgacaacttatgcactga	aagataattggaacgtaagtctgtacc	NM_145518.2 , NM_001160038.1 , NM_001160039.1	3,33
IR5358	Sdhb	42	ctggtggaacggagacaagt	gcgttcctctgtgaagtcgt	NM_023374.3	3,38
IR5359	Sdhc	68	cacctgaatgctcagctttgt	ttccagaaccgctccatct	NM_025321.3	3,3
IR5360	Sdhd	3	tctgctcttggggctgat	cccatgaacgtagtcggtaac	NM_025848.3	3,3
IR5365	Pfkl	42	attgaccggcatggaaag	aagcccagcctctgaacc	NM_008826.4	3,22
IR5641	Cpt1a	56	cttcaatacttcccgcatcc	gcctctgtggtacacgacaat	NM_013495.2	3,3
IR5972	Il4ra	97	gagtggagtcctagcatcacg	cagaggcaggagatggtga	NM_001008700.3	3,34
IR6088	Me1	16	cagaggccctgagtatgacg	ccgattggcaaaatcttcaa	NM_008615.2, NM_001198933.1	3,49
IR6089	Pgd	48	aaagatccgggacagtgct	gagcaaagacagcttctccaa	NM_001081274.2	3,13
IR6090	Esrra	45	gacctctggcagtagctgga	tggcgtacagcttctcaggt	NM_007953.2	3,36
IR6227.2	Ccl22	53	gccaggactacatccgtca	cggttcttgacggttatcaaa	NM_009137.2	3,42
IR6228	Il4i1	45	gggcctcaatcggacttc	caatcctgttatctgcctcca	NM_010215.3	3,6
IR6229	Idh1	77	tcagaaggtgacatacatggtaca	ttggaaggaactgtgtgcaa	NM_010497.3, NM_001111320.1	3,26
IR6230	Ptgs1	76	actggtggatgccttctctc	tctcgggactccttgatgac	NM_008969.4	3,18
IR6231.2	Alox15	10	gagattgggttgcaaggtg	gatgaagtggcaagcctga	NM_009660.3	3,8
IR3659.3	Actb	56	aaggccaaccgtgaaaagat	gtggtacgaccagaggcatac	NM_007393.3	3,33
IR3669.2	Gapdh	80	tgtccgtcgtggatctgac	cctgcttcaccaccttcttg	NM_008084.2	3,55
